# Automatically tailored exercise app training is feasible, usable, and safe for people with paraplegia: a parallel mixed methods pilot study

**DOI:** 10.1186/s13102-026-01801-x

**Published:** 2026-06-12

**Authors:** Janika Bolz, Johanna Marseille, Adrian Löscher, Rainer Muhl, Hans-Georg Predel, Claudio Perret

**Affiliations:** 1 Institute of Cardiovascular Research and Sports Medicine, German Sport University Cologne, Am Sportpark Müngersdorf 6, Cologne, 50933 Germany; 2 BG Clinic Tübingen, Tübingen, Germany; 3 Swiss Paraplegic Research, Nottwil, Switzerland; 4 Faculty of Health Sciences and Medicine, University of Lucerne, Lucerne, Switzerland

**Keywords:** Paraplegia, Exercise, Mobile app, Algorithm, mhealth, Feasibility, Usability

## Abstract

**Background:**

Mobile exercise apps for people with paraplegia that offer accessible and customized exercise programs have the potential to promote fitness and health in this population. However, there is a lack of suitable exercise apps that do not require the support of healthcare professionals for exercise progression. The ParaGym app was designed to automatically tailor and progress the training intervention using an algorithm. This study aimed to assess the feasibility and safety of the exercise program, as well as the usability of the app to determine whether the app should be further developed.

**Methods:**

Twenty-three participants with chronic paraplegia (mean age 48 ± 13 years, 15/8 male/female) performed three app-delivered exercise sessions per week over six weeks. A mixed-methods approach was used to comprehensively assess feasibility, usability, and safety based on semi-structured interviews, exercise diaries, adherence and retention rates, the System Usability Scale (SUS), and reported adverse and severe adverse events. Interviews were analyzed using structured qualitative content analysis.

**Results:**

Interview themes comprised satisfaction, adherence and motivation, training sessions and exercises, technical functionality, ease of use, and perceived safety. Participants expressed overall satisfaction with the app, as affirmed by the intention to continue use, an adherence rate of 70%, and a retention rate of 88%. Exercises were overall suitable and feasible, supported by easily understood videos and descriptions. Areas for improvement included frequent transitions between wheelchair and floor exercises and insufficient time to change positions during timed exercises. The mean SUS score was 84 ± 11, indicating good usability. This was confirmed during interview sessions. No adverse events occurred, and participants reported feeling safe during the training.

**Conclusions:**

The app-delivered exercise sessions were overall feasible, and the app was easy to use. Further development is indicated and should prioritize practical considerations, such as grouping floor exercises, integrating additional safety considerations, and providing features for the user to further personalize the program. A future marketable and publicly available version for wheelchair users of all fitness levels can make a valuable contribution to low-threshold leisure opportunities.

**Trial Registration:**

German Clinical Trials Register (DRKS00030370), October 25, 2022.

**Supplementary Information:**

The online version contains supplementary material available at 10.1186/s13102-026-01801-x.

## Introduction

Individuals with spinal cord injury (SCI) show considerably lower activity levels than non-disabled persons or people with other chronic conditions such as stroke [1]. Low activity levels and sedentary behavior are associated with a higher risk for cardiovascular diseases, diabetes, obesity, and other secondary diseases [2]. This especially affects people with an SCI, who depend on a wheelchair for mobility [3]. Regular physical activity such as exercise [4] is an effective means to reduce these risks and to increase cardiovascular fitness, quality of life, and independence [3, 5–10]. However, engaging in regular physical activities is complicated for this underrepresented population. They encounter constant barriers, including inadequate accessibility, transportation obstacles, and a lack of knowledge on how and where to exercise, as well as disability-specific knowledge among exercise instructors [11–14].

Home-based exercise programs delivered via mobile exercise apps have the potential to overcome these barriers: they do not require changing location and can guide participants step by step through entire exercise programs [15, 16]. Furthermore, home-based exercise interventions have been shown to significantly increase cardiorespiratory fitness, health-related quality of life, and shoulder pain in individuals with paraplegia [5, 6, 17, 18]. Thus, app-based exercise programs may have the potential to achieve similar effects, promote participation in regular physical activity, and thereby lead to an active and healthy lifestyle [19, 20]. However, there is only one publicly available mobile exercise app for this population [21]. Two additional exercise apps have been presented in the research [22, 23]. However, the integrity of the single released app [21] is questionable, as it was last updated in 2019 and does not provide details about its privacy practices [24]. The linked website for this product has no mention of their app [25]. Neither of the other two apps in development can be used without the support of healthcare professionals, and they are not available on the public app market [22, 23]. However, it is questionable whether healthcare professionals can provide this support in the long term in addition to their regular patient care, limiting the app training’s sustainability. Additionally, attaining and maintaining maximum independence is central to persons with disabilities and drives their exercise participation [10, 26, 27]. Consequently, there is a lack of mobile exercise apps that offer accessible leisure-time exercise programs tailored to the needs of people with paraplegia and that can be used independently of medical care but may also complement it.

Research on relevant exercise app features has highlighted the importance of good usability and customization [28–32]. To our knowledge, there is no app available that meets these needs. The implementation of exercise programs for individuals with paraplegia furthermore requires injury-related safety considerations, for example the provision of extensive information on injury-related risks, such as autonomic dysreflexia [3, 33, 34]. The safety evaluation of an intervention is considered an important aspect, especially in pilot trials [35].

The novel ParaGym mobile exercise app prototype (hereafter referred to as the “ParaGym app”) was developed to provide people with paraplegia with an accessible, guided, and safe exercise program that can be done in the comfort of their own home. We aimed to create a leisure-time exercise program that is customized and can be carried out independently of healthcare professionals’ support, complementing their usual care. The ParaGym app uses an algorithm to automatically customize the exercise program for each user, considering their level of impairment, available equipment, and fitness level.

It is the overarching aim of this study to determine whether the prototype should be further developed into a market-ready version, based on user perceptions on the feasibility, usability, and safety of the ParaGym app and the exercise program, as well as the anticipated feasibility of implementing proposed changes.

## Methods

### Description of the ParaGym App

**Fig. 1:**
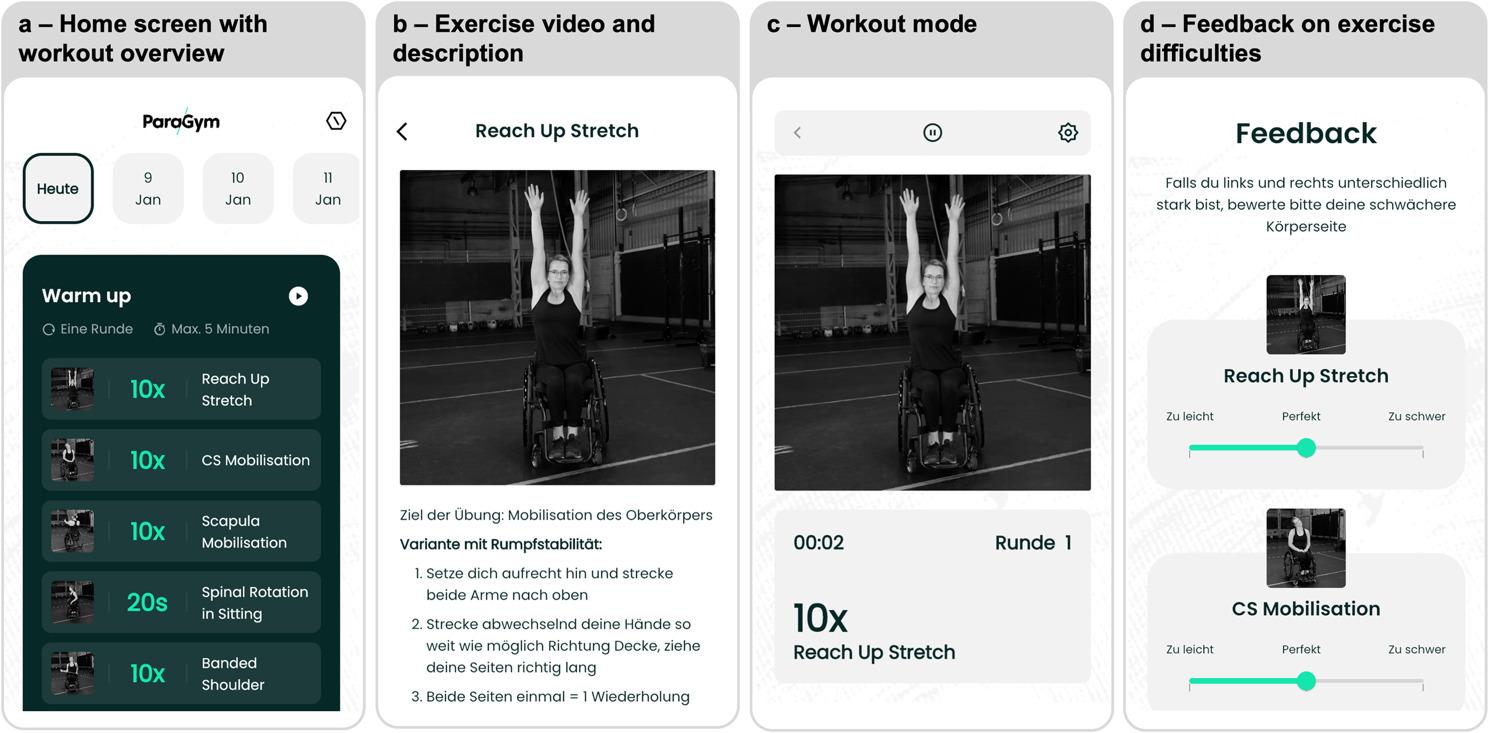
**a-d** Overview ParaGym app

The development of the ParaGym app was based on the Kernwerk^®^ Functional Fitness app [36] for non-disabled persons [37]. A more detailed description of the app’s development process, its content, and the algorithm’s functionality is provided in the study protocol [37]. When first starting the app, users must confirm they have read and understood the comprehensive safety advice. This safety advice can be accessed in the settings at any time. This app uses an algorithm to automatically tailor the exercise program to the user [37–39]. The user sets up the app by selecting physical capacity filters based on their level of physical impairment (e.g., impaired trunk stability, ability to extend the knee) and available equipment (e.g., arm crank ergometer). Each exercise was attributed with the required equipment and physical capacity, and the difficulty level. Similar exercises were then linked to each other. This enables the algorithm to find a possibly more suitable exercise if filters do not apply to the originally planned one. The exercise pool was developed with and validated by the target group and physical therapists in this field [38]. Based on these user settings, the app automatically adjusts a predetermined workout shown on the home screen (Fig. 1a). Selecting one of the exercises displays the corresponding video and description (Fig. 1b). Daily workouts targeting various exercise goals are suggested. This includes the improvement in wheelchair mobility or trunk stability. After pressing the play button to begin a workout, generic safety questions (e.g., “Have you been to the toilet?”) are displayed, followed by additional instructions on what to keep in mind while performing the exercises (e.g., “Pull your shoulders down, away from your ears”). A 10-second countdown begins each workout, after which the exercise video and the number of repetitions are displayed (Fig. 1c). Users can proceed to the next exercise by simply touching the screen. After completing the last exercise, users are asked to provide feedback on the level of difficulty of each performed movement (Fig. 1d). Exercises rated as too easy will be automatically replaced with a more advanced exercise, and vice versa. Users can also access the exercise catalog in the settings, view instructions, and block specific exercises. After blocking an exercise, a similar, potentially more suitable one is integrated into this user’s workouts.

### Study design

**Fig. 2:**
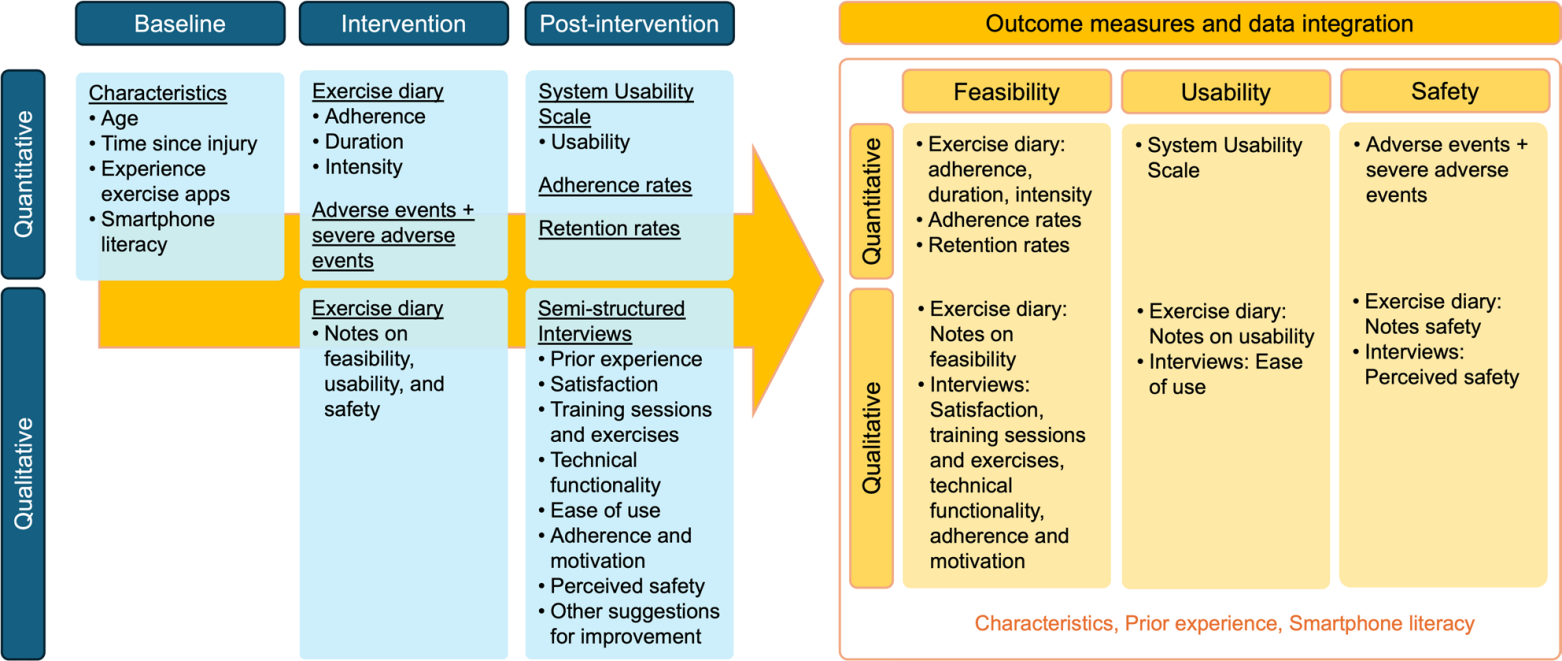
Parallel mixed methods study design and outcome measures

A parallel mixed methods approach [40–42] was used to comprehensively assess the feasibility, usability, and safety of the app prototype. The study design and outcome measures are presented in Fig. 2. This design was chosen to gain a better understanding of each outcome by integrating qualitative (QUAL) and quantitative findings (quan) [42–44]. Another purpose was to minimize social desirability bias. Quantitative data were collected to quantify and operationalize the outcome measures, and to validate qualitative findings. They may also facilitate comparability to other studies and to potential future studies evaluating future versions of the ParaGym app. Qualitative data were collected to validate quantitative findings (convergence function/triangulation), to provide a deeper understanding of them, to assess related aspects (complementary function), and to explain them (expansion function) [42, 45]. Quantitative and qualitative data were collected simultaneously, giving more weight to qualitative findings (QUAL + quan) [42].

The research team consisted of exercise scientists and therapists, as well as a physician (HGP). Authors do not strongly identify with one particular research paradigm. The design of the study and the interpretation of its results were influenced by a pragmatic approach (e.g., what do we need to learn about the ParaGym app? ) as well as by transformative and constructivist views. This is reflected in the authors’ career choices, as they share the common goal of supporting their clients or patients to improve their health and well-being, as well as in the overarching project goal of creating an accessible exercise program. Furthermore, the project adopted a user-centered approach by involving the target group in several phases of the project and within the research team (e.g., co-creation workshops, requirement analysis survey, author with lived experience) [37, 38, 46].

The trial was registered with the German Clinical Trials Register (No. DRKS00030370) prior to study commencement (2022/10/25) and was approved by the ethics committee of the German Sport University (GSU; No. 180/2021) in accordance with the Declaration of Helsinki [47]. All data was collected between January 2023 and June 2023. This trial is reported according to the Consolidated Criteria for Reporting Qualitative Research (COREQ) [48], the Standards for Reporting Qualitative Research (SRQR) [49], and Good Reporting of A Mixed Methods Study (GRAMMS) [50]. Respective checklists are provided in Additional file 1.

At baseline, potential participants were invited to the GSU and the BG (*Berufsgenossenschaft*, English: occupational insurance association) clinic Tübingen (BGC) for a medical screening to ensure the absence of medical contraindications (medical clearance) [3, 33, 34]. This included resting echocardiography and the Physical Activity Readiness Questionnaire for Everyone (PAR-Q+) [51]. Enrolled participants received an app demonstration [22, 23] by either JB, AL, or RM and were provided with the app, a user manual, safety advice, two resistance bands (TheraBand^®^, The Hygenic Corporation, Akron, OH, USA), and an exercise diary (Additional file 2). Participants were asked to perform an approximately 45-minute exercise program at home, delivered by the app three times per week for six weeks. After the intervention, participants revisited the study sites to complete the SUS questionnaire and return the exercise diary. Additionally, semi-structured interviews were conducted.

### Intervention

The program included a warm-up, two workout parts, and a cool-down, including 5–7 exercises per part. It comprised strength endurance, flexibility, and wheelchair mobility exercises, targeting upper as well as lower extremities. Exercise guidelines for people with SCI recommend strength training at least two times per week for major muscle groups at moderate to vigorous intensity [3, 52]. As strengthening exercises were only one component of this program, the frequency was set at three sessions per week [37]. Two sample workouts – one for users with residual leg function and one for users without – are included in Additional file 2.

### Participants

The aim was to recruit 30 participants for the feasibility trial based on recommendations for pilot, usability, and safety trials [53–55] and studies with comparable aims and methods [22, 56]. Participants at the GSU were recruited openly via social media and through flyer distribution at rehabilitation centers (purposive sampling). The challenge of recruiting a sufficient sample size in studies targeting people with SCI is well known [35]. To increase the recruitment rate, the GSU has partnered with the BGC. The BGC recruited among their patients (convenience sampling) between November 2022 and April 2023. Inclusion criteria for participants comprised an age range of 18 to 67 years, chronic paraplegia (> 1 year), sufficient German language skills, the ability to provide informed consent, and access to a smartphone. The criteria for exclusion consisted of all medical contraindications to exercise, such as manifest cardiovascular disease, uncontrolled diabetes or hypertension, acute infection or inflammation, mental conditions that could compromise informed consent or interfere with study results, and ongoing substance abuse [57]. Participants were also excluded if they were high-performance athletes [37]. All participants gave written informed consent prior study participation.

### Outcomes

Semi-structured interviews based on an interview guide (Additional file 3) were conducted to assess feasibility, usability, and perceived safety. This approach allowed researchers the flexibility to add follow-up questions and to maintain a conversational flow while ensuring the coverage of all relevant domains [58]. JB developed the interview guide by reviewing similar feasibility trials investigating mHealth interventions and retracting any relevant domains from questionnaires, interview guides, and frameworks used in these studies [22, 59–73]. It was pilot tested and discussed with an experienced colleague. The pilot test did not result in any changes. The final guide comprised nine domains. Of these, seven addressed feasibility ((1) overall experience/satisfaction, (2) app functionality, (3) technical functionality, (4) communication, (5) perceived effects and usefulness, (6) adherence and motivation, and (7) recommendation) one addressed usability ((8) ease of use), and one addressed safety ((9) perceived safety). The interview was offered to all participants without stopping when potential data saturation was reached to gain sufficient data richness and maximize information power. Hereby, all participants had the opportunity to share their individual experiences. Participants at the GSU were interviewed face-to-face in a meeting room, while those at the BGC were interviewed online via Webex (version 43, Cisco Systems, Inc., San José, CA, USA). All interviews were conducted in German, carried out by the same person (JB), and audio recorded anonymously with the interviewees’ consent. Face-to-face interviews at the GSU were recorded using the Voice Memos app (iPhone SE (2020), Apple, Cupertino, CA, USA), and online interviews were recorded using the Webex recording function. JB was a female fellow researcher trained in interviewing techniques and content analysis. She is a PhD candidate in sports science and the lead investigator for this study. One participant had their spouse present during the interview. The few field notes taken were redundant with the audio recording and were therefore not analyzed. At the beginning of the interview, all participants were encouraged to speak honestly about challenges with the app. Participants were informed that constructive criticism and honesty were the best ways to contribute to enhancing the app prototype. This was intended to limit social desirability bias and convey a genuine interest in participants’ actual experiences. Some participants had learned that JB had a family member with paraplegia, which might have conveyed a genuine interest in the research topic.

#### Feasibility

Feasibility was assessed qualitatively based on the interview domains 1–7 (Additional file 3). Additionally, participants were asked to take notes on the feasibility of the performed exercise sessions using the exercise diary (Additional file 2). Insights gained from the interview were complemented by quantitative data retrieved from the exercise diaries, including duration and intensity (Borg Ratings of Perceived Exertion Scale (RPE)) [74] of each completed exercise session. Due to technical capacity constraints and data protection requirements, it was not possible to integrate a feature into the app that would enable the analysis of user behavior. Therefore, adherence rates were obtained from exercise diary records. Participants were considered adherent to the program if they completed at least 66.7% of the exercise sessions, equivalent to two out of three sessions per week (≥ 12 total sessions), and in accordance with exercise guidelines [3, 37, 52, 62]. Additionally, retention rates were assessed. Feasibility was assumed if (1) the retention rate was at least 75% [37, 75], (2) the adherence rate was at least 66% [37], and (3) participants were overall satisfied with the app-delivered training and the app’s components. This complementary approach was chosen to gain a deeper understanding of the users’ experience and their perception of the appropriateness and suitability of, for example, the exercises as well as the duration and intensity of the sessions.

#### Usability

Usability was assessed qualitatively based on the interview domain 8, feedback provided in the exercise diaries (Fig. 2; Additional file 3 (Interview Guide)), and quantitatively using the SUS, which is a widely applied and robust tool [23, 61, 62, 66, 72, 76, 77]. The SUS is a 10-item Likert scale ranging from 1 (“strongly disagree”) to 5 (“strongly agree”), with total scores ranging from 0 to 100. The app was considered usable (1) if the overall SUS score is at least 70 as proposed by Bangor et al. (2008) [77] and (2) if the SUS score is congruent with interviewees’ feedback on “Ease of use” (convergence function) [42]. Furthermore, the qualitative results are intended to provide a deeper understanding of the participants’ experiences and reasons that may have influenced the usability ratings and, consequently, the respective SUS scores (complementary function and expansion function) [42].

#### Safety

The safety of this home-based exercise program was assessed qualitatively based on the participants’ perceived safety reported in the interviews (domain 9) and exercise diaries (Fig. 2; Additional file 3), and quantitatively based on the occurrence of adverse and severe adverse events [65, 66, 78]. Participants were instructed to report any adverse or severe adverse events to investigators within 24 h [37] and complete a report form. These were classified using the Spinal Adverse Events Severity System version (SAVES-V2) scale. This complementary approach [42] was chosen to examine whether participants felt safe during the execution of all exercises and to identify factors that might have contributed to the occurrence and non-occurrence of adverse and severe adverse events.

### Analyses

To ensure confidentiality, participants’ names were pseudonymized using IDs (e.g., P12). These were used during the analyses and for reporting all data.

#### Quantitative analyses

SPSS Statistics 29 (IBM, Armonk, NY, USA) was used to descriptively analyze demographic data, duration, and intensity of the exercise sessions, and SUS scores. It was also used to conduct normality tests using the Shapiro-Wilk test and group comparisons using the Kruskal-Wallis test. Demographic data, with the exception of time since injury, were distributed normally. All continuous data were reported as means and standard deviations (SD) regardless of distribution, to improve readability without compromising informative value. Skewed and nominal data were presented using medians and interquartile ranges (IQR) as well as absolute and relative frequencies, respectively. SUS scores for all participants (*n* = 23) and for all subgroups were normally distributed except for the interviewee subgroup (*n* = 18). SUS scores were reported as mean (SD) to allow for comparison. An independent-samples Kruskal-Wallis test was used to explore differences between subgroups. The significance level for this test was set at *p* ≤ 0.05. Any missing data were not imputed.

#### Qualitative analyses

Audio recordings were transcribed verbatim by JB and JM using f4transkript (version 8.2.0, audiotranskription, dr. dresing & pehl GmbH, Marburg, Deutschland) following the transcription guide (Additional file 3) [79, 80]. JM, a female student assistant and exercise scientist, had not engaged in the study prior to the coding process and was therefore unbiased. She was trained in transcription and coding techniques for this study. JB conceptualized and developed the app’s content and was deeply familiar with the app [37]. There was no relationship to any of the participants prior to study commencement. All interaction was limited to study matters. Subsequently, the transcripts were imported into MAXQDA Analytics Pro 24 (Release 24.11.0, VERBI GmbH, Berlin, Germany). The structured qualitative content analysis approach by Kuckartz and Rädiker (2022; 2023) [22, 80–82] with a hierarchical category frame was used to analyze each interview. This approach was chosen for three reasons: First, it provides detailed, easy-to-follow, and therefore comprehensible step-by-step instructions that improve transparency. Second, according to this approach, only the original text is used during the analysis without paraphrasing the coded text segments. This might limit potential subjective interpretations of these segments at an early stage of the analysis. Third, it is compatible with the MAXQDA software.

After familiarizing with the text, JB deductively formed a-priori main categories based on the interview guide, literature [22, 56, 61, 67], and knowledge of the interview content. The main categories were rearranged, differentiated, and defined by employing them in three interviews. In a first coding process, these three transcripts were coded separately by JB and JM. Subsequently, discrepancies were discussed until consensus was reached. The categories were further refined to ensure clear differentiation from one another. Category definitions and coding guidelines are available in the codebook (Additional file 3). Subcategories were inductively derived from the transcripts and documented in the codebook (Additional file 3). The coding and consensus processes were repeated until the categories were sufficiently defined and differentiated; this occurred when the codes were assigned consistently by both coders. The remaining transcripts were coded by JB. Participants confirmed the credibility and accuracy of the preliminary qualitative content analysis results in the context of the project’s closing event [81]. Coded segments were compared between cases for each category by deriving a case-code-matrix from the MAXQDA software (qualitative case comparison) [45]. Because some coded segments spanned multiple paragraphs, all coded segments were paraphrased to capture the core meaning of each statement. Each outcome was presented in a separate Excel sheet (Additional file 4). Within the manuscript, direct quotes were translated to English and reported with reference to the participant ID and the quoted transcript section (e.g., P12, Sect.  98) to substantiate findings and add trustworthiness. Original quotes are available in Additional file 4.

#### Mixed methods integration

Quantitative and qualitative data were analyzed and reported separately. JB compiled all data by adding the quantitative results to the case-code-matrix to build a side-by-side joint display separately for each outcome (Additional file 4) [41, 43–45]. In the next step, she integrated the data at the interpretation level using a weaving approach [44].

## Results

**Fig. 3:**
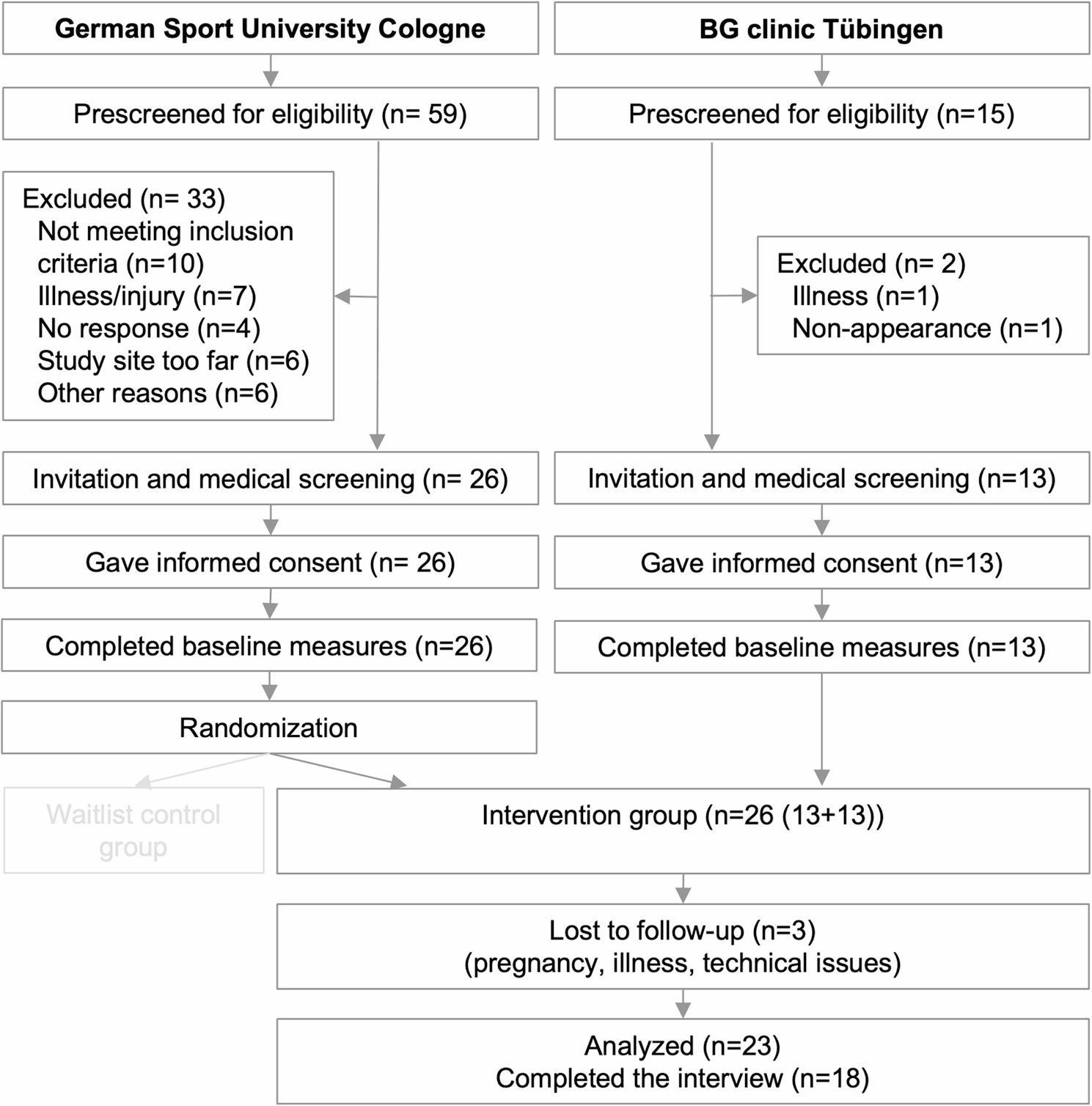
Participant flow. BG: Berufsgenossenschaft; Note: the waitlist control group was part of a larger trial and will not be further discussed

**Fig. 4:**
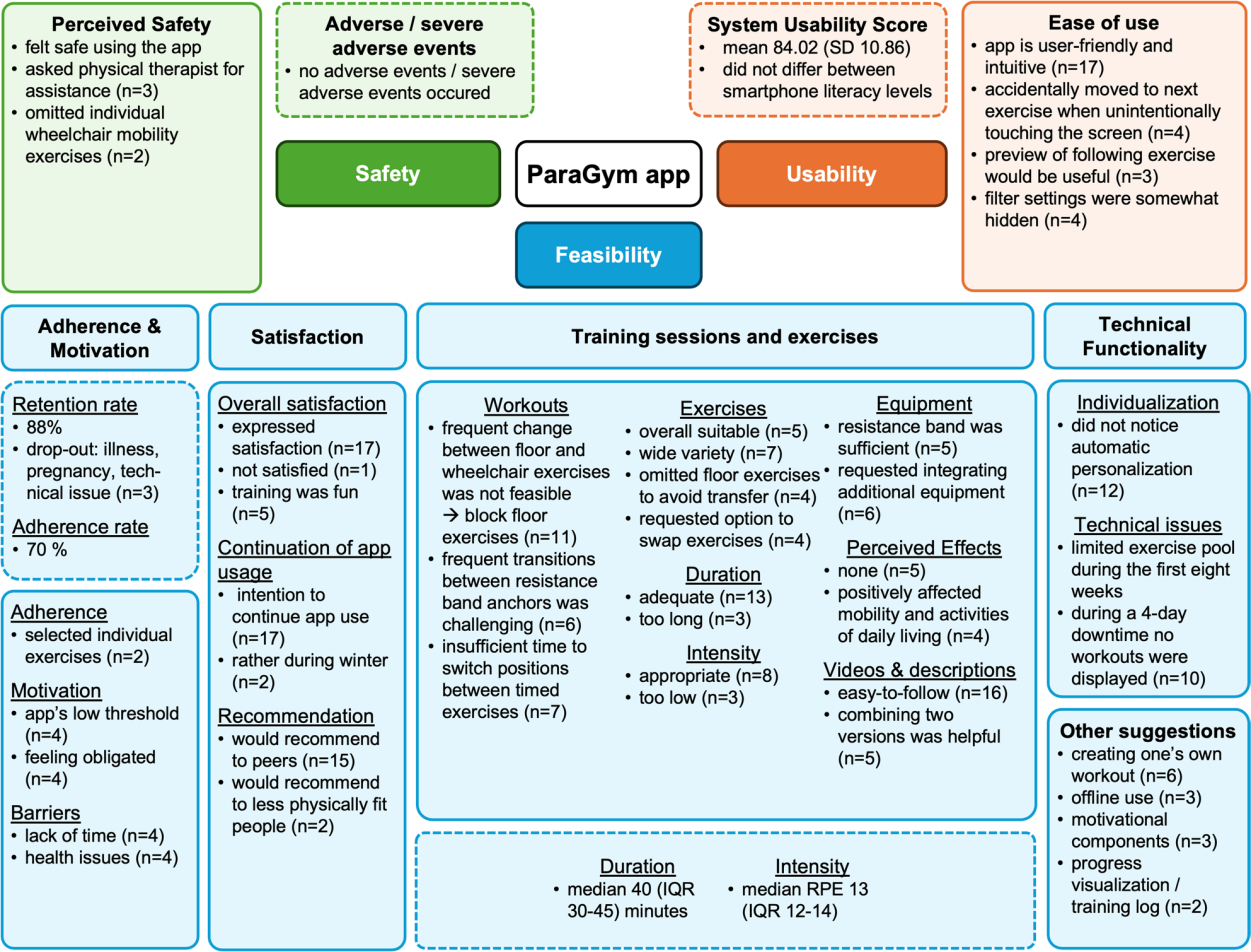
Main qualitative (solid box border) and quantitative findings (dotted box border). n: number of users who made this statement

This study was part of a larger trial, which included a waitlist control group. Thus, for the sake of completeness, this group is presented in the participants flow chart (Fig. 3) but will not be mentioned further. Eighteen participants agreed to the interview. The average interview duration was 38:07 (SD 08:37) minutes. The final category system encompasses eight main and 15 subcategories (Additional file 3). An overview of the main qualitative and quantitative findings for each outcome, main category, and subcategory is presented in Fig. 4.

### Participants

Table 1 displays the participants’ characteristics, including ratings of their smartphone literacy on a scale from 1 to 6 based on the German school grading system (“How experienced are you at using smartphones?”: 1 = excellent/very experienced; 2 = good; 3 = alright; 4 = adequate; 5 = poor; 6 = insufficient/very inexperienced). Participants varied in age (range 18–65 years), time since injury (range 1.5–39 years), lesion level (range from third thoracic segment (T3) motor complete to T12 motor incomplete), and smartphone literacy (range 1–4). Five participants reported prior experience with exercise apps. Interviews revealed that they had only used devices that track distance and duration, for example (P06, P36, P41) or exercise apps before becoming paraplegic (P25, P210). P15 added that they personally had no experience using exercise apps “*because most of them are not meant for wheelchairs or for movements made out of a wheelchair*” (Sects.  1–2).

**Table 1:** Participants’ characteristics

Characteristics	Total (*N* = 23)	Completed interview (*n* = 18)
Age (years), mean (SD)	47.51 (13.05)	49.78 (12.07)
Gender, n (%)		
Men	15 (65.2)	11 (61.1)
Women	8 (34.8)	7 (38.9)
Non-Binary	0 (0)	0 (0)
TSI, mean (SD)	13.48 (13.98)	15.86 (14.94)
Lesion level, n (%)		
T1-T6	7 (30.4)	5 (27.8)
T7- S5	16 (69.6)	13 (72.2)
Lesion completeness, n (%)		
motor complete	19 (82.6)	15 (83.3)
motor incomplete	4 (17.4)	3 (16.7)
Experience with exercise apps, n (%)		
Yes	5 (21.7)	5 (27.8)
No	18 (78.3)	13 (72.2)
Smartphone literacy, n (%)		
1 - excellent	6 (26.1)	5 (27.8)
2 - good	10 (43.5)	8 (44.4)
3 - alright	5 (21.7)	4 (22.2)
4 - adequate	2 (8.7)	1 (5.6)
5 - poor	0 (0)	0 (0)
6 - insufficient	0 (0)	0 (0)

*Abbreviations*: *N/n* Sample size, *S* Sacral segment, *T* Thoracic segment, *TSI* Time Since Injury

### Feasibility

#### Adherence and retention rates (quan)

Twenty-six individuals were provided with access to the exercise app (Fig. 3). Three participants dropped out due to pregnancy (*n* = 1), illness (*n* = 1), and technical issues (*n* = 1) resulting in a sample size of *N* = 23 and a retention rate of 88%, which exceeded the predefined cut-off value of 75%. According to the exercise diaries, 16 out of 23 participants completed at least twelve exercise sessions using the app, leading to an adherence rate of 70%; this exceeded the predefined cut off value of 66%. On average, participants completed 15.1 (SD 7.7) exercise sessions, equivalent to 2.5 sessions per week.

#### Adherence and motivation: adherence, motivation, and barriers (QUAL)

One participant admitted to occasionally filling out the exercise diary retrospectively (P38). Two participants (P25, P201) indicated that they sometimes created personalized workouts, selecting individual exercises from the exercise catalog rather than adhering to the app’s workout suggestions. Factors motivating participants to use the app included the low threshold (i.e., customized, guided, and “ready-to-go” predetermined short-duration trainings) (P06, P36, P38, P212), the opportunity to remain active during winter months (P23, P201, P206), and feeling obligated by their study participation (P06, P23, P35, P210). Barriers included lack of time due to alternative activities, such as physical therapy (P06, P12, P38, P201, P206), illness (P23; P38, P15, P41), and a general preference for exercising outdoors or in groups (P35, P38, P206).

#### Satisfaction: overall satisfaction, continuation of app usage, and recommendation

Seventeen out of 18 participants were quite satisfied with the app and would like to continue using it. Five participants indicated that their continued use of the app would be conditional on several factors. These included seasonal use, preferring winter app training (P201, P207), its value as a source of inspiration (P36, P201), the addition of certain features, such as the ability to create custom workouts and add new exercises (P38) or to log training activities (P206). Just one participant (P35) responded that they prefer guided group training and have no intention to continue using the app. Participants appreciated the app’s low threshold: “*I really liked the fact that it gives you ideas for exercising and strengthening your muscles using the simplest of means*,* without the need for lots of equipment or a gym. It’s easy to do anytime*,* anywhere*,* as long as you have internet access.”* (P15, Sect.  6). Five participants stated using the app was fun (P12, P14, P23, P39, P212). They also appreciated having everything in one place (P06, P201), independent of the time and place (P06, P38). “*When you’re in the hospital*,* you always end up with 500 or 600 pieces of paper telling you what you have to do*,* how you have to do it*,* and so on. But if you have an app where everything is super easy*,* where you just check in every now and then for stretching exercises and sports activities*,* it was just ideal*,* and I think I’ll use it more again in the winter.*” (P201, Sect.  47).

Fifteen participants stated that they would recommend the app to peers or have already recommended it. Two of them would recommend the app to people who have more severe impairments or are less fit than themselves (P38, P201), have no access to therapy or a gym (P35), are rather technologically proficient (and therefore are more likely to have their phone at hand) (P207), or upon request (206).

### Training sessions and exercises

#### Workouts

Eight participants expressed their satisfaction with the structure of the workouts (P12, P14, P23, P25, P36, P200, P206, P41). *“In general*,* I thought the warm-up and the four blocks were really cool*,* because then […] it was like a small unit that was manageable and where you didn’t say*,* ‘Oh*,* another 20 minutes or half an hour.’ Instead*,* they were blocks that made you think*,* ‘Come on*,* you can do it.’*” (P200, Sect.  42).

The main criticism pertained to the order of the exercises. Frequent transfers between floor and wheelchair exercises drew negative attention (P06, P14, P15, P23, P25, P39, P40, P200, P207, P210, P212), as did transitions between different resistance band anchors (P12, P23, P25, P35, P207, P210). This was considered time-consuming and inconvenient. Completing the exercises in one block is one possible solution (P06, P14, P15, P23, P25, P200, P207). “*What I also find difficult is*,* when there were transitions between floor and wheelchair in a set or series so to say. I solved this for myself by simply doing the floor exercises one after another at the end or at the beginning.”* (P06, Sect.  38).

Another point of criticism was the short countdown before and during timed exercises (e.g., stretching exercises). Users stated this countdown provided insufficient time to get into position or transition from previous exercise positions (P12, P14, P23, P25, P36, P201, P41). This could be easily solved by either extending the countdown or allowing users to begin it manually (P36, P200).

#### Perceived effects

Five participants linked the absence of perceived effects to the duration of the intervention or the insufficient intensity of the sessions (P12, P35, P36, P38, P41). Four participants confirmed that the app helped them to increase their physical activity level (P39, P41, P207, P210), while five participants reported that it did not due to their already high physical activity level (P06, P15, P25, P40, P206). Four participants reported that the exercises became easier over time (P15, P23, P25, P40), three felt that they had become fitter (P25, P200, P212), and two participants reported increased confidence when performing wheelchair mobility drills (P06, P212). Four users described the intervention as having an impact on their mobility and activities of daily living (P14, P200, P207, P212) “*Well*,* mobility – you can see I’m not the slimmest person – but mobility in terms of being able to bend down to your feet or sideways to the wheels and*,* in everyday life*,* to pick things up*,* um*,* to move around. That has already made quite a difference*.” (P200, Sect.  10). Furthermore, perceived positive impacts on their well-being (P14, P39, P200, P206, P210, P212), shoulder and back pain, shoulder mobility (P14, P207), and spasticity (P14) were reported.

#### Equipment

Exercising exclusively using a resistance band and bodyweight was considered sufficient by five participants (P14, P23, P36, P38, P212). Users appreciated the versatility of the band (P06, P14, P23, P41) and how it can be taken anywhere (P15, P36, P200, P210, P212). “*I really liked that. I actually thought*,* ‘Wow*,* it’s impressive what you can do with this little thing.*” (P23, Sect.  20). Six participants, on the other hand, suggested integrating additional equipment, such as dumbbells (P06, P12, P15, P35, P40, P210), or a cable machine (P15). Further remarks included difficulties finding a suitable anchor point (P12, P212), challenges comparing how exercises were performed across different days when using a resistance band (P06), and occasional discomfort when gripping the band (P06).

#### Exercises

Overall, most exercises were suitable and feasible (P12, P38, P201, P206, P210). One participant indicated that only a few exercises were sufficiently demanding but still considered them suitable for less athletically trained people (P38). After initially being disappointed by the limited variety of exercises due to a technical issue, seven participants reported being impressed by the variety once the issue was resolved (P06, P14, P23, P25, P36, P38, P40). While P06 and P212 found the wheelchair mobility exercises helpful even after several years of wheelchair use, P38 considered them too easy. Four participants omitted the floor exercises to avoid transfer and expressed their preference for alternative exercises that could be performed in the wheelchair, as well as the option to swap exercises (P15, P35, P200, P206). The integration of more stretching exercises was requested by three participants (P06, P40, P212). One user was negatively surprised that most exercises focused on everyday movements rather than compensatory movements, which they felt were not sufficiently addressed (P38). P207, on the other hand, stated “*Well*,* you also do movements that you don’t usually do*.” (Sect.  34). P15 noticed “*And then*,* when you do the exercises conscientiously*,* you realize*,* ‘Damn*,* there’s a deficit’. Most of the time*,* the exercises revealing your weaknesses are the ones contrary to your everyday movements.*” (Sect.  64).

#### Duration and intensity (quan)

Two participants did not record session durations. The median duration of the exercise sessions was 40 (IQR 30–45) minutes for all participants (*n* = 21) and 40 (IQR 35–45) minutes for interviewees (*n* = 17), as recorded in the exercise diaries.

Three participants did not record session intensities. The median intensity of the exercise sessions was RPE 13 (IQR 12–14) for all participants (*n* = 20) and RPE 13 (IQR 12–14) for interviewees (*n* = 17) based on the exercise diaries.

#### Duration and intensity (QUAL)

Interviewees reported taking between 30 and 60 min to complete an exercise session, which they thought was adequate (P06, P12, P15, P23, P25, P35, P36, P38, P39, P200, P201, P206, P207) and easy to integrate into the daily routine (P06, P15, P23, P38). On the contrary, three participants felt that the duration was too long (P14, P210, P212).

All interviewees, except three who felt it was too low (P35, P38, P40), considered the intensity appropriate. After addressing the previously mentioned technical issue, more exercises became available in the app. Four participants noticed that this led to an increased workout intensity (P12, P14, P25, P36).

#### Exercise videos and descriptions

Both the exercise videos and descriptions were easy to understand and to follow (P06, P12, P14, P15, P23, P25, P35, P36, P39, P40, P41, P201, P206, P207, P210, P212). Demonstrating and describing two alternative versions of the same exercise in the video and accompanying text was deemed helpful by five participants (P06, P23, P207, P210, P212). However, one participant would have preferred the option to select one version and hide the other (P207). “*I always started doing the ones without trunk stability*,* and every now and then tried to do the ones with it. I mean the ones with trunk stability*,* to make it a bit more challenging for me. That actually worked better over time*.” (P23, Sect.  32) One participant reported confusion caused by the video switching between the two exercise versions and overlooked the second description (P12). Two participants noted that certain details, such as the exact path of the resistance band or scapular movements, were difficult to discern. They suggested adding a visual cue in the video, referring to the description or increasing the contrast (P12, P14). Furthermore, four participants proposed that unilateral exercises should be demonstrated on both sides in the videos (P15, P23, P36, P40). Showing videos during the workout was considered helpful for emulating the pace of exercises and reduce the risk of performing them incorrectly (P25, P39, P206). One participant appreciated the authenticity of the videos, which displayed models with high and low levels of paraplegia (P25).

### Technical functionality

#### Technical issues

**Fig. 5:**
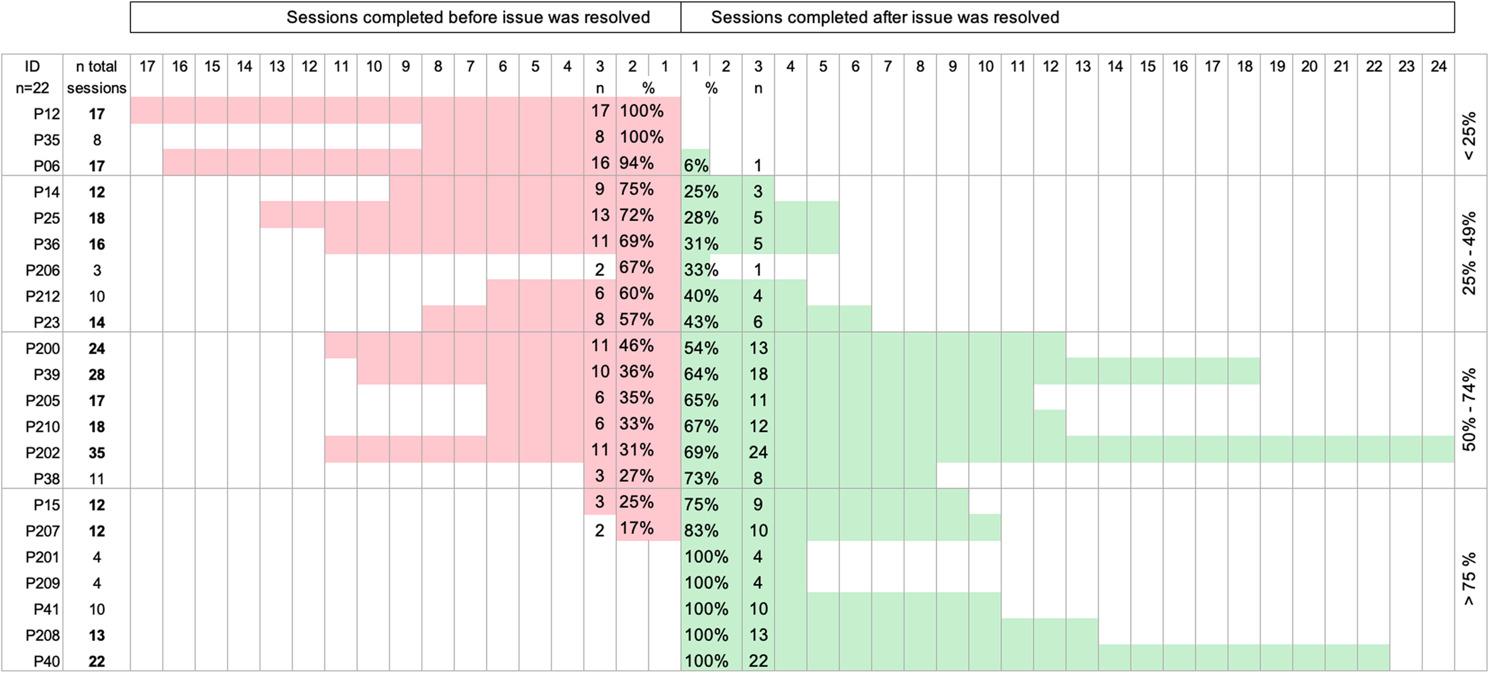
Number of participants and extent to which they were affected by the limited exercise pool issue (n = 22)

During the first days of the intervention period, a technical issue prevented exercise videos from loading. Also, users were not returned to the home screen upon completing their workout. However, this issue was resolved within one day and affected only the first participant enrolled (P12). One participant was unable to register for the app and therefore withdrew from the study. During the first eight weeks of the intervention period, the algorithm integrated only 25 basic exercises (i.e., the easiest version of each exercise) out of a total of 67 exercises into the workouts due to a technical error. These basic exercises could not be blocked by the user. Following a four-day downtime, during which workouts could not be displayed (P06, P15, P25, P35, P36, P39, P40, P200, P206, P210), all exercises and workouts became available in the app. As participants began the intervention consecutively over a six-month period, only a subset of them was severely affected by this issue. The extent to which participants were affected varied among them, depending on the total number of sessions and the proportion of those completed before and after the issue was resolved (Fig. 5). P203 commenced the 6-week intervention two weeks before the issue was solved and completed 22 sessions but did not record any dates. Therefore, it can be assumed that approximately 66% of the sessions were completed after the issue was solved. In total, 14 of 23 participants (61%) completed more than half of the exercise sessions after the error was corrected. The issue did not appear to affect program adherence (Fig. 5). Thirteen of 16 participants who were involved in the study before and after solving the issue recorded session intensities. The median session intensity reported by these participants was RPE 13 (IQR 12–14) before and after the issue was resolved.

#### Individualization

Two participants appreciated the ability to filter exercises by physical ability and block individual exercises (P36, P201). “*Appropriate exercises are also presorted for you*,* so that’s really good.*” (P201, Sect.  65). When asked whether they had noticed any personalization of the program in response to their filter settings or post-session feedback, twelve participants answered negatively. However, two of these reported that the exercises were still feasible (P39, P212). Three users, on the other hand, felt that the filter settings had an impact on the program (P12, P23, P41). P14 and P25 reported that the filters began working after the downtime. Four participants mentioned too high repetition counts, up to 100 repetitions (P12, P14, P35, P41).

### Usability

#### System usability score (quan)

The mean SUS score was 84 (SD 10.9, range 55-97.5) for all participants (*N* = 23) and 84.9 (SD 11.7, range 55-97.5) for the interviewees (*n* = 18). Participants who rated their smartphone literacy as excellent (*n* = 6) had an average SUS score of 88.8 (SD 7.2). Those who rated it as good (*n* = 10), alright (*n* = 5), or adequate (*n* = 2) had an average SUS score of 81.3 (SD 11.9), 82.5 (SD 13.8), and 87.5 (SD 7.1), respectively. There were no significant differences between these subgroups (*p* ≥ 0.22).

Participants who had completed > 75% of sessions before the issue causing a limited exercise pool was resolved (*n* = 3) had a mean SUS score of 80 (SD 9), those who had finished 51–75% (*n* = 6) had a mean SUS score of 88.8 (SD 8.8), those who had finished 26–50% (*n* = 7) had a mean SUS score of 83.9 (SD 10.2), and those who had finished 25% or less (*n* = 7) had a mean SUS score of 81.8 (SD 14.2). There were no significant differences between these subgroups (*p* ≥ 0.16).

All mean scores, therefore, exceed the cut-off value of 70. Thus, the app is considered usable [77]. The distribution of ratings for each item is displayed in Table 2, showing that all item ratings have a tendency toward one of the scale endpoints, while the ratings for items 5 (“Integration”) and 6 (“Inconsistency”) tend toward the middle.

**Table 2:** Distribution of system usability scale ratings (*N* = 23), n (%)

Item	Strongly disagree				Strongly agree
	1	2	3	4	5
I think that I would like to use this system frequently.	2 (9)	4 (17)	2 (9)	6 (26)	9 (39)
I found the system unnecessarily complex.	17 (74)	4 (17)	1 (4)	1 (4)	0 (0)
I thought the system was easy to use.	0 (0)	1 (4)	1 (4)	7 (30)	14 (61)
I think that I would need the support of a technical person to be able to use this system.	22 (96)	1 (4)	0 (0)	0 (0)	0 (0)
I found the various functions in this system were well integrated.	0 (0)	2 (9)	9 (39)	7 (30)	5 (22)
I thought there was too much inconsistency in this system.	5 (22)	10 (43)	4 (17)	4 (17)	0 (0)
I would imagine that most people would learn to use this system very quickly.	0 (0)	0 (0)	1 (4)	7 (30)	15 (65)
I found the system very cumbersome to use.	16 (70)	7 (30)	0 (0)	0 (0)	0 (0)
I felt very confident using the system.	1 (4)	1 (4)	1 (4)	6 (26)	14 (61)
I needed to learn a lot of things before I could get going with this system.	20 (87)	3 (13)	0 (0)	0 (0)	0 (0)

#### Ease of use (QUAL)

All participants rated the app as generally user-friendly (P12, P15, P25, P38, P40, P41, P200, P207, P212) or even very user-friendly and intuitive to use (P06, P14, P23, P36, P39, P201, P206, P210) with the exception of one participant (P35). This participant could not find the start button for the workout and therefore used the training overview. An app tutorial would have supported this participant in better understanding the app and its functions. The workout overview on the home screen was deemed useful (P14, P35), and the simple design was noted as appealing (P25). Two participants did not know that they could block exercises (P38, P207). P207 suggested the integration of this in addition to the possibility of swapping exercises in the workout overview. This participant intentionally did not start the workout mode because they preferred using the overview during the workout. Four participants accidentally skipped exercises during the workout by unintentionally touching the screen (P15, P39, P200, P201). Possible solutions proposed by three users include: touching and holding the screen for several seconds to move to the next exercise (P15), integrating a button (P201), or controlling the app using a smartwatch (P39). Additionally, three users mentioned that a preview of the next exercise during the current workout would be helpful for preparation (P12, P39, P200). Four users mentioned during the interview that the filter settings were somewhat hidden or that they could not find them (P14, P25, P35, P40). P40 suggested moving these from the user profile to the general settings, which were easy to find.

### Safety

#### Adverse and severe adverse events (quan)

No adverse or severe adverse events were reported.

#### Perceived safety (QUAL)

None of the participants stated feeling unsafe during the training. The demonstration of two exercise alternatives within the same video helped them to select a version that is appropriate for their individual capabilities (P15), and the safety advice was deemed helpful and sufficient (P35, P39). Two participants asked a health care professional for feedback on exercise execution (P41, P207), while eight users did not require any assistance (P12, P14, P15, P23, P36, P201, P206, P210). *“But it’s not like I’ve ever thought*,* no*,* that’s too risky for me right now. Or am I scared that I’ll hurt myself or whatever*,* yeah.”* (P206, Sect.  58) Two participants did not feel comfortable performing individual wheelchair mobility drills without assistance and therefore chose not to complete them (P25, P212). Three participants sought support while performing wheelchair mobility exercises, asking a physical therapist for assistance, or leaned against a wall (P06, P36, P39).

### Further suggestions for improvement

The most frequently requested additional features included the opportunity to further personalize the program by creating personalized individual workouts (P15, P25, P38, P201, P206, P210) and by integrating the option to choose between different workout durations (P210). Future versions of the app should provide the ability to use it offline (P06, P15, P200), to block or manually switch exercises, and include exercises for more athletically advanced users (P40, P210). Three participants proposed adding motivation components (P23, P200, P212), such as spoken or written prompts during the workout (e.g., “let’s go, you can do it”, P200, Sect.  12). Two users would appreciate the option to pair the app with their smartwatch to track repetitions, for example (P200, P201), and the opportunity to log training sessions (e.g., exercises, duration, intensity) to better visualize their progress (P200, P206).

Further suggestions included making the app accessible for people with tetraplegia (P06), offering different training programs with specific aims (e.g., learning wheelchair transfers) (P15, P210, P212), and providing additional health-related information, such as dietary advice (P200) or information on shoulder pain (P39). Four participants appreciated the app’s simplicity and its clear focus on training, and had no further suggestions (P14, P36, P41, P207).

## Discussion

This manuscript reports qualitative perceptions and quantitative measures of the feasibility, usability, and safety of the novel algorithm-based ParaGym app in a heterogeneous sample of participants with paraplegia. Qualitative findings on the one hand indicated an overall satisfaction supported by the intention of continued use, willingness to recommend the app to peers, a high retention rate (88%), as well as a moderate adherence rate (70%), though, on the other hand, revealed the considerable need for further personalization options and technical revision. The app was considered user-friendly according to the interviewees’ perception of ease of use and the average SUS score of 84 (SD 11). No adverse or severe adverse events occurred during the study, and all participants reported feeling safe while using the app.

### Feasibility

The adherence rate of (70%) is similar to that reported by Froehlich-Grobe et al. (2022) [83] regarding the self-guided exercises during the first 1–2 months of the “Wheelchair on wheels internet intervention” (WOWii) trial and exceeds that reported by Wilroy et al. (2021) [84] for the “music-to-movement” (M2M) trial. The 12-week M2M-intervention included prerecorded exercise videos that were not tailored to the individual user, and exercise adherence reported for the WOWii-intervention decreased to 43% after four months. This is in line with our findings, according to which participants were more likely to use the program more often if they could further customize it, for example, by creating their own workouts. This underscores the pressing need for additional program customization options [28–32] and for users to perceive personal value in using the app [30] to facilitate adherence. In this study, adherence averaged 15 total completed exercise sessions, but varied greatly between participants, ranging from four to 35 sessions. While seven participants completed fewer than twelve sessions, five exceeded the prescribed 18 total sessions. Qualitative findings provide insight into the potential reasons for this. The most frequently mentioned barriers were intervention-unrelated health issues (e.g., cold) and lack of time due to work, frequent physical therapy sessions, or the general preference to exercise outdoors (e.g., go handbiking) or in groups. These barriers were also observed by Hoevenaars et al. (2021) [22] and reflect real-world conditions. While this app-delivered program overcomes structural barriers, such as inaccessibility of buildings or lack of transport, it does not tackle barriers on a personal level, such as lack of time and interest [11–13, 85], which has been considered a primary barrier for the continued use of mobile health apps [86]. Facilitators to use the app included its low threshold, meaning the provision of a customized and guided program that is independent of time and location, using portable, low-cost equipment. This confirms that app-based training is indeed a means to overcome the structural barriers mentioned above. Two of the participants who exceeded the prescribed volume, attributed their enthusiasm for the app to the low threshold and linked perceived improvement in general well-being to the app training. The latter, again, affirms the importance of perceived personal value in using the app as a driving factor [30].

In addition to the factors facilitating adherence, users’ satisfaction with the app may have been attributed to their overall content with the variety and feasibility of the exercises, the duration, and the intensity of the sessions. Quantitative analyses could not confirm the users’ perception that the intensity increased after the technical issue limiting the exercise pool was solved. It is noteworthy that the median intensity of RPE 13 and the average number of completed sessions per week are in accordance with current exercise guidelines for strength training in individuals with SCI [3, 52]. Lastly, comprehensible, easy-to-follow, and authentic exercise descriptions and videos that allowed users with partially innervated trunk musculature to select the more appropriate exercise version may also have contributed to the high level of satisfaction. This represents an improvement over the exercise component of the WHEELS app, where users experienced greater difficulty identifying suitable exercises and creating a training program despite the wide range of available options [22]. The integration of exercise videos represented by individuals with SCI as models is considered a useful tool to enhance engagement and relatability [29, 87].

The retention rate of 88% is consistent with that in 8- to 16-week (partly) staff-guided exercise interventions for individuals with SCI (81–84%) [40, 83] and even exceeds that in 8–12 week self-guided programs or intervention phases for individuals with SCI or Parkinson’s disease (60–75%) [5, 22, 65, 83]. In contrast to Hoevenaars et al. (2021) and Landers and Ellis (2020), who reported attrition due to non-compliance and dissatisfaction, drop-out reasons in the present study were not associated with the intervention program. However, this intervention only lasted six weeks. Longer intervention periods may be associated with higher attrition [5, 22, 65].

Issues participants were dissatisfied with, and suggestions for improvement were linked to (1) practical considerations, (2) the need for more options for customization, (3) technical issues, and (4) motivational aspects:

(1) Practical considerations involve ensuring that the user does not have to rely on the help of others to carry out exercises and arrange equipment as described by Stavric et al. (2023) [30]. Concretely, this encompasses the grouping of floor exercises into a single block to avoid transfers, extending the countdown before timed exercises or by implementing an option to start the exercise timer manually, and providing separate videos for each side to allow users to adjust their position at an appropriate pace. Additionally, the need for frequent adjustments of the band’s fixation could be decreased by incorporating more exercises using other equipment, such as dumbbells, and consequently reducing reliance on resistance bands only.

Another issue relates to (2) the need to further customize the app by implementing options for the user to personalize the program themselves and therefore giving them greater control over the program [30]. The importance of this feature has been highlighted by several authors who investigated the perception of individuals with SCI and relevant stakeholders regarding mobile self-management and exercise apps [28–31, 86]. Concretely, participants discussed the option to create one’s own workout and to block and swap exercises.

(3) Technical issues and functionality were related to the earlier-discussed limited exercise pool, high repetitions counts, and the perception that filter options and feedback did not have an impact on the workouts. As this app was still in a prototype stage, the occurrence of technical errors was not unexpected. Most of these issues were resolved within a few days. Several participants reported not perceiving any personalization. This may be explained by three factors: First, the small selection of equipment and the low difficulty level of exercises may have limited automatic exercise progression and load increase. Second, the impression that the equipment and physical capacity filters were not functioning may be due to most exercises relying on the resistance band only, combining exercises requiring trunk stability with those that do not, and not all workouts containing exercises targeting lower limbs. Third, the inability to block certain exercises was not a technical error but a predefined system feature. This applied to basic exercises only, as at this stage, no simpler alternative exercise exists in the app. By developing the algorithm and implementing filters as suggested by Nataletti and colleagues (2024) [29], we were able to address the limitation described by Hoevenaars et al. (2021) [22], in which exercises could not be further personalized due to software constraints. To address these issues and meet users’ needs, future versions should incorporate a wider variety of exercises suitable for advanced users and that complement everyday movements. Additionally, available, accessible, and affordable equipment such as dumbbells should be integrated [29, 88–90].

(4) Requested features that have the potential to motivate users and improve accountability have been reported repeatedly by individuals with SCI and healthcare professionals. This includes the integration of reminders [28–30, 86], training logs and progress tracking [28–30, 86], and social and educational resources [28–31, 86]. In contrast, the integration of reward systems has been frequently reported by other authors [28–30, 86, 87], but only mentioned by one participant in this study. A reminder function, training log, a social feed, and a knowledge base are already integrated into the Kernwerk^®^ exercise app [36] for non-disabled individuals, which served as the basis for the development of the ParaGym app but were not included in the present app due to its prototype status. Thus, implementing these features in future versions of the ParaGym app may be feasible. To address feedback from participants who valued the app’s simplicity, future versions should maintain a primary focus on the workouts while offering additional features in a more subtle, customizable manner by making them optional and easy to disable.

### Usability

Good usability is considered one of the most important features of an app for (potential) users with SCI to initiate and maintain its use [28, 29, 31, 32, 87]. According to the 7-point adjective rating scale introduced by Bangor et al. (2008) [77], a mean SUS score of 84 reflects not only a good usability (≥ 72.8), but nearly an excellent one (≥ 85.6). Authors [22, 60, 62, 66] investigating mobile exercise apps for adults of advanced age or with chronic conditions reported SUS scores ranging from 59 [22] to 86 [60]. In contrast to the WHEELS app [22], participants found the ParaGym app easy to use (91%) and negated the statement that the app was “cumbersome to use” [76] (100%). It is worth noting that the average SUS score was above 80, regardless of the level of smartphone literacy or the extent to which participants were affected by the issue, causing the limited exercise pool. This emphasizes the app’s usable and intuitive design despite its prototype stage. Participants’ perceptions of ease of use were consistent with these quantitative findings and revealed factors that may have contributed to the high SUS score: They described the app as generally user-friendly, and highlighted the workout overview and clean, simple app design. Areas for improvement appear to be reflected in the distribution of ratings across items 5 (“Integration”) and 6 (“Inconsistency”), and may include difficulties handling the app – such as accidentally skipping exercises – and with finding the filter settings, which were somewhat difficult to locate. In contrast to Hoevenaars et al.’s (2021) findings, these difficulties are not likely to be related to a lack of motivation. In addition, the lack of desired additional app features may have contributed to these ratings. Integrating these could enhance users’ general experience and contribute to the improvement of these ratings.

### Safety

For the successful implementation of an app, the safety of the exercise program is an important prerequisite [30, 87]. All participants reported feeling safe while using the app and some noted that the exercise videos helped to reduce the risk of performing the exercises incorrectly. This was supported by the non-occurrence of adverse and severe adverse events. These results are similar to those reported for other home-based exercise interventions for people with chronic conditions [18, 65, 66]. However, these findings should not be misinterpreted to mean that there is no residual risk, as the qualitative findings shed some light on factors that contributed to the non-occurrence of adverse and severe adverse events. This included omitting exercises participants did not feel comfortable with and asking for support. However, this shows that the participants were aware of the risk of falling and took safety precautions on their own initiative.

Although a residual risk of a sporting injury will always remain with the user, the following measures could be taken to improve the programs safety: (1) integration of an in-app questionnaire to determine whether users should seek medical clearance before using the app, for example the American College of Sports Medicine’s (ACSM) pre-participation health screening [40, 91]; (2) adding visual cues to the videos to use a mirror and refer to the description as suggested by some participants; (3) integration of a feature which enables users to give feedback on their experience with individual exercises including safety concern similar to the in-app questionnaires used by Landers & Ellis (2020) [65].

### Limitations

The recruitment aim of 30 participants was not reached, resulting in a smaller sample size. However, according to Faulkner (2003), only 20 participants were sufficient to detect at least 95% of the usability problems, with a mean of 98.4% (SD 1.6). Furthermore, we are confident that the essential aspects of the app’s feasibility, usability, and safety were captured, as critical comments in the exercise diaries were consistent with interview feedback, and major themes were reported by multiple participants. Additionally, the main areas for improvement aligned with the findings in studies on required features of mobile self-management and exercise apps. A selection bias is unlikely an issue, as the sample varied widely in age, time since injury, level of impairment, and prior experience, and several participants expressed a general lack of interest in app-based training. This study included individuals of employable age between 18 and 67 years old. Recruitment challenges could be mitigated by removing the upper age limit.

We cannot rule out social desirability bias despite the measures taken to minimize it. This bias may have differed between study sites: participants at the GSU were tested by JB both at baseline and post-intervention and were interviewed face-to-face, whereas participants at the BGC met JB online for the first time. In addition to presenting preliminary results to the participants during the closing event, interview transcripts should be returned to them for correction to ensure credibility and accuracy.

Self-reporting adherence, duration, and intensity using the exercise diary constitutes a serious limitation to the reliability of these outcomes. One participant indicated that the exercise diary was sometimes completed retrospectively, which may have introduced a recall bias. Unfortunately, it was not possible to retrieve app use data, which would have provided more reliable adherence data. Adherence and retention rates may have been positively influenced by participants’ sense of commitment. To address this limitation, ass long-term adherence and motivation to use the app, and improve the sustainability of physical training, longer intervention periods and post-intervention follow-up assessments similar to Froehlich-Groebe et al. (2022) [83] should be implemented in future studies.

Feasibility measures were based on an extensive literature review. However, employing benchmarks (e.g., “at least 75% of participants reported they were satisfied with XY.” [75]) might have enhanced comprehensibility and comparability and strengthened the meaningfulness of this outcome. The algorithm’s functionality was considerably constrained by the limited number of exercises and the selection of equipment, which, in turn, restricted the progression of the training and the adaptation to the needs of athletically advanced users. Consequently, the algorithm’s functionality cannot be evaluated.

Although no adverse or severe adverse events occurred and participants reported feeling safe, limited conclusions can be drawn about general and long-term safety, e.g., the occurrence of overuse injuries or falls. A residual risk of injury remains with the user and cannot be eliminated. However, the participants have demonstrated that they were aware of the risks and have taken responsibility for their own safety.

No limitation of one method associated with the presence of the other method could be identified.

### Implications

Implications relate to the further development of the app and to potential trials assessing future versions. Practical considerations include grouping floor exercises and adding a button to manually start timed exercises. Grouping of floor exercises could be achieved by unlinking them from wheelchair exercises. As a result, the algorithm cannot substitute floor exercises for wheelchair exercises and vice versa, meaning that the exercises are no longer mixed within the workout, and floor exercises can be intentionally scheduled in blocks. Additionally, another filter option should be added that allows users to enable and disable floor exercises. Disabling floor exercises should result in the app providing an alternative workout part using wheelchair exercises.

Another major implication is the integration of further options to customize the app, including the creation of users’ own workouts and the ability to block and swap exercises. In addition, users should be able to adjust the repetition count, a feature already integrated into the Kernwerk^®^ app.

To allow for an evaluation of the algorithm’s functionality and for proper exercise progression, a greater number and variety of exercises, including more advanced exercises and those targeting the lower limbs, as well as more equipment such as dumbbells, should be integrated. A longer alpha testing phase may improve the detection and resolution of technical issues prior to the intervention phase. Additionally, the validation of the algorithm should involve exercise experts experienced in spinal cord injury rehabilitation.

Future versions of the app should furthermore implement features that motivate users to use the app and adopt a physically active lifestyle, such as reminders [29, 87]. This trial focused on overcoming structural barriers but neglected personal barriers, such as a lack of motivation. The further development of the app should therefore consider factors that facilitate the uptake and maintenance of physical activity, for example, by applying behavior change techniques and frameworks [87]. Future trials should employ a longer intervention period and a post-intervention retention phase. Adherence rates should be drawn from app usage data to improve reliability. Furthermore, wheelchair users beyond those living with paraplegia may benefit from the opportunity to exercise at home. Therefore, a more diverse cohort should be targeted.

To improve usability, a button to move to the next exercise or a smartwatch interface and an exercise preview should be integrated. The filter settings should be moved to general settings to improve discoverability.

Safety implications include integrating visual cues into exercise videos and in-app questionnaires and feedback options.

## Conclusions

The ParaGym app prototype already presents good usability for individuals who are not tech-savvy and the provision of an overall suitable exercise program, features that were identified as most important by individuals with SCI and relevant stakeholders [28–32, 46, 86]. The intervention appeared to be safe. However, there is still a fundamental need for further customization and safety precautions. Given that features such as motivational and educational components or blocking and swapping individual exercises have already been successfully integrated into the Kernwerk^®^ app, which served as the basis for the ParaGym app, the implementation of most of the proposed changes is likely feasible. Therefore, the app should be further developed to a market-ready product and has the potential to become the first publicly available mobile exercise application for wheelchair users and people with mobility impairments that offers automated progression.

## Supplementary Information

Additional file 1: PDF; Reporting guidelines checklists: COREQ, SRQR; GRAMMS.

Additional file 2: PDF; Exercise diary and sample workout.

Additional file 3: PDF; Interview analysis material: Interview Guide, Transcription Rules, Codebook, Coding Guideline.

Additional file 4: Excel (.xlsx); Joint display of qualitative and quantitative results.

## Data Availability

The datasets used and analyzed during the current study are available from the corresponding author on reasonable request. All coded segments and respective paraphrases can be found in Additional file 4.

## Abbreviations

ACSM: American College of Sports Medicine
BCG: BG Clinic Tübingen
COREQ: Consolidated Criteria for Reporting Qualitative Research
GRAMMS: Good Reporting of a Mixed Methods Study
GSU: German Sport University Cologne
IQR: Interquartile range
PAR-Q+: Physical Activity Readiness Questionnaire for Everyone
RPE: Ratings of Perceived Exertion
SCI: Spinal cord injury
SD: Standard Deviation
SRQR: Standards for Reporting Qualitative Research
SUS: System Usability Scale

## References

[1] van den Berg-Emons RJ, Bussmann JB, Stam HJ. Accelerometry-Based Activity Spectrum in Persons With Chronic Physical Conditions. Arch Phys Med Rehabil. 2010;91:1856–61. 10.1016/j.apmr.2010.08.018. 21112426 10.1016/j.apmr.2010.08.018

[2] Evans N, Wingo B, Sasso E, Hicks A, Gorgey AS, Harness E. Exercise Recommendations and Considerations for Persons With Spinal Cord Injury. Arch Phys Med Rehabil. 2015;96:1749–50. 10.1016/j.apmr.2015.02.005. 26198424 10.1016/j.apmr.2015.02.005

[3] Tweedy SM, Beckman EM, Geraghty TJ, Theisen D, Perret C, Harvey LA, et al. Exercise and sports science Australia (ESSA) position statement on exercise and spinal cord injury. J Sci Med Sport. 2017;20:108–15. 10.1016/j.jsams.2016.02.001. 27185457 10.1016/j.jsams.2016.02.001

[4] World Health Organization [WHO]. WHO guidelines on physical activity and sedentary behaviour. Geneva: World Health Organization; 2020. https://apps.who.int/iris/bitstream/handle/10665/336656/9789240015128-eng.pdf?sequence=1&isAllowed=y. 33369898

[5] Mulroy SJ, Thompson L, Kemp B, Hatchett PP, Newsam CJ, Lupold DG, et al. Strengthening and Optimal Movements for Painful Shoulders (STOMPS) in Chronic Spinal Cord Injury: A Randomized Controlled Trial. Phys Ther. 2011;91:305–24. 10.2522/ptj.20100182. 21292803 10.2522/ptj.20100182

[6] Nightingale TE, Rouse PC, Walhin J-P, Thompson D, Bilzon JLJ. Home-Based Exercise Enhances Health-Related Quality of Life in Persons With Spinal Cord Injury: A Randomized Controlled Trial. Arch Phys Med Rehabil. 2018;99:1998–2006. 10.1016/j.apmr.2018.05.008. 29902472 10.1016/j.apmr.2018.05.008

[7] van der Scheer J, Martin Ginis K, Ditor DS, Goosey-Tolfrey V, Hicks A, West C, et al. Effects of exercise on fitness and health of adults with spinal cord injury. A systematic review. Neurology. 2017;89:736–45. 10.1212/WNL.0000000000004224. 28733344 10.1212/WNL.0000000000004224

[8] Hicks AL, Martin KA, Ditor DS, Latimer AE, Craven C, Bugaresti J, et al. Long-term exercise training in persons with spinal cord injury: effects on strength, arm ergometry performance and psychological well-being. Spinal Cord. 2003;41:34–43. 10.1038/sj.sc.3101389. 12494319 10.1038/sj.sc.3101389

[9] Nash MS, Groah SL, Gater DR, Dyson-Hudson TA, Lieberman JA, Myers J, et al. Identification and Management of Cardiometabolic Risk after Spinal Cord Injury. J Spinal Cord Med. 2019;42:643–77. 10.1080/10790268.2018.1511401. 31180274 10.1080/10790268.2018.1511401 PMC6758611

[10] Hanson CS, Nabavi D, Yuen HK. The Effect of Sports on Level of Community Integration as Reported by Persons With Spinal Cord Injury. Am J Occup Ther. 2001;55:332–8. 10.5014/ajot.55.3.332. 11723975 10.5014/ajot.55.3.332

[11] Fekete C, Rauch A. Correlates and determinants of physical activity in persons with spinal cord injury: A review using the International Classification of Functioning, Disability and Health as reference framework. Disabil Health J. 2012;5:140–50. 10.1016/j.dhjo.2012.04.003. 22726854 10.1016/j.dhjo.2012.04.003

[12] Cowan RE, Nash MS, Anderson KD. Exercise participation barrier prevalence and association with exercise participation status in individuals with spinal cord injury. Spinal Cord. 2013;51:27–32. 10.1038/sc.2012.53. 22584283 10.1038/sc.2012.53

[13] Williams TL, Smith B, Papathomas A. The barriers, benefits and facilitators of leisure time physical activity among people with spinal cord injury: a meta-synthesis of qualitative findings. Health Psychol Rev. 2014;8:404–25. 10.1080/17437199.2014.898406. 25211208 10.1080/17437199.2014.898406

[14] Martin Ginis KA, Ma JK, Latimer-Cheung AE, Rimmer JH. A systematic review of review articles addressing factors related to physical activity participation among children and adults with physical disabilities. Health Psychol Rev. 2016;10:478–94. 10.1080/17437199.2016.1198240. 27265062 10.1080/17437199.2016.1198240

[15] McMillan DW, Astorino TA, Correa MA, Nash MS, Gater DR. Virtual Strategies for the Broad Delivery of High Intensity Exercise in Persons With Spinal Cord Injury: Ongoing Studies and Considerations for Implementation. Front Sports Act Living. 2021;3:703816. 10.3389/fspor.2021.703816. 34423292 10.3389/fspor.2021.703816 PMC8377288

[16] Lai B, Wilroy J, Young H-J, Howell J, Rimmer JH, Mehta T, et al. A Mobile App to Promote Adapted Exercise and Social Networking for People With Physical Disabilities: Usability Study. JMIR Form Res. 2019;3:e11689. 10.2196/11689. 30888325 10.2196/11689 PMC6444218

[17] Nightingale TE, Walhin J-P, Thompson D, Bilzon JLJ. Impact of Exercise on Cardiometabolic Component Risks in Spinal Cord–injured Humans. Med Sci Sports Exerc. 2017;49:2469–77. 10.1249/MSS.0000000000001390. 28753161 10.1249/MSS.0000000000001390 PMC5704648

[18] Kesiktaş FN, Kaşıkçıoğlu E, Paker N, Bayraktar B, Karan A, Ketenci A, et al. Comparison of the functional and cardiovascular effects of home-based versus supervised hospital circuit training exercises in male wheelchair users with chronic paraplegia. Turk J Phys Med Rehab. 2021;67:275–82. 10.5606/tftrd.2021.6533. 10.5606/tftrd.2021.6533 PMC8606995 34870113

[19] Parker K, Uddin R, Ridgers ND, Brown H, Veitch J, Salmon J, et al. The Use of Digital Platforms for Adults’ and Adolescents’ Physical Activity During the COVID-19 Pandemic (Our Life at Home): Survey Study. J Med Internet Res. 2021;23:e23389. 10.2196/23389. 33481759 10.2196/23389 PMC7857525

[20] Plow M, Golding M. Using mHealth Technology in a Self-Management Intervention to Promote Physical Activity Among Adults With Chronic Disabling Conditions: Randomized Controlled Trial. JMIR Mhealth Uhealth. 2017;5:e185. 10.2196/mhealth.6394. 29196279 10.2196/mhealth.6394 PMC5732326

[21] Bernard RM, Seijas V, Davis M, Volkova A, Diviani N, Lüscher J, et al. Self-Management Support Apps for Spinal Cord Injury: Results of a Systematic Search in App Stores and Mobile App Rating Scale Evaluation. JMIR Mhealth Uhealth. 2024;12:e53677. 10.2196/53677. 39700493 10.2196/53677 PMC11695972

[22] Hoevenaars D, Holla JFM, te Loo L, Koedijker JM, Dankers S, Houdijk H, et al. Mobile App (WHEELS) to Promote a Healthy Lifestyle in Wheelchair Users With Spinal Cord Injury or Lower Limb Amputation: Usability and Feasibility Study. JMIR Form Res. 2021;5:e24909. 10.2196/24909. 34379056 10.2196/24909 PMC8386360

[23] Bizzarini E, Chittaro L, Frezza M, Polo M, Malisan C, Menosso R, et al. A mobile app for home-based exercise in spinal cord injured persons: Proposal and pilot study. Digit Health. 2022;8:205520762110707. 10.1177/20552076211070724. 10.1177/20552076211070724 PMC8819755 35140978

[24] Monster Hub. Neuro Therapy. 2019. https://apps.apple.com/de/app/neuro-therapy/id1479833318?l=en-GB. Accessed 16 Apr 2026.

[25] Combined Wellness Solution. https://www.cwsau.com.au. Accessed 16 Apr 2026.

[26] World Health Organization, World Bank, editors. World report on disability. Geneva, Switzerland: World Health Organization; 2011.

[27] Pebdani RN, Leon J, Won DS, deLeon RD, Dy CJ, Forsyth R, et al. It Helps Me With Everything: A Qualitative Study of the Importance of Exercise for Individuals With Spinal Cord Injury. Top Spinal Cord Injury Rehabilitation. 2022;28:176–84. 10.46292/sci21-00049. 10.46292/sci21-00049 PMC9009197 35521059

[28] Bendixen RM, Fairman AD, Karavolis M, Sullivan C, Parmanto BA, User-Centered, Approach. Understanding Client and Caregiver Needs and Preferences in the Development of mHealth Apps for Self-Management. JMIR Mhealth Uhealth. 2017;5:e141. 10.2196/mhealth.7136. 28951378 10.2196/mhealth.7136 PMC5635231

[29] Nataletti S, Banerjee A, Macaluso R, Prokup S, Jayaraman A, Wong AWK. Developing a mobile exercise program for individuals with Spinal Cord Injury: Stakeholder perceptions of app features and implementation determinants. Disabil Health J. 2024;17:101667. 10.1016/j.dhjo.2024.101667. 38964938 10.1016/j.dhjo.2024.101667

[30] Stavric V, Saywell NL, Kayes NM. Perceptions of a self-guided web-based exercise programme for shoulder pain after spinal cord injury: A qualitative study. Spinal Cord. 2023;61:238–43. 10.1038/s41393-023-00877-3. 36702921 10.1038/s41393-023-00877-3 PMC10070182

[31] Mortenson WB, Singh G, MacGillivray M, Sadeghi M, Mills P, Adams J, et al. Development of a Self-Management App for People with Spinal Cord Injury. J Med Syst. 2019;43:145. 10.1007/s10916-019-1273-x. 31011881 10.1007/s10916-019-1273-x

[32] Singh G, MacGillivray M, Mills P, Adams J, Sawatzky B, Mortenson WB. Patients’ Perspectives on the Usability of a Mobile App for Self-Management following Spinal Cord Injury. J Med Syst. 2020;44:26. 10.1007/s10916-019-1487-y. 10.1007/s10916-019-1487-y 31828440

[33] Zehr EP. Evidence-based risk assessment and recommendations for physical activity clearance: stroke and spinal cord injury. Appl Physiol Nutr Metab. 2011;36:214–31. 10.1139/h11-055. 10.1139/h11-055 21800943

[34] Rosa SA, Westcott WL. Physical Fitness Programming for Individuals with Spinal Cord Injury: Paraplegia and Tetraplegia. Strength Cond J. 2010;32:19–21. 10.1519/SSC.0b013e3181f3d5ae.

[35] Lammertse D, Tuszynski MH, Steeves JD, Curt A, Fawcett JW, Rask C, et al. Guidelines for the conduct of clinical trials for spinal cord injury as developed by the ICCP panel: clinical trial design. Spinal Cord. 2007;45:232–42. 10.1038/sj.sc.3102010. 17179970 10.1038/sj.sc.3102010 PMC4106695

[36] Kernwerk^®^. Kernwerk^®^. https://welcome.kernwerk.de/en. Accessed 10 May 2026.

[37] Bolz J, Löscher A, Muhl R, Badke A, Predel H-G, Perret C. Feasibility, Usability, and Safety of ParaGym, an Intelligent Mobile Exercise App for Individuals With Paraplegia: Protocol for a Pilot Block-Randomized Controlled Trial. JMIR Res Protoc. 2023;12:e45652. 10.2196/45652. 37204855 10.2196/45652 PMC10238958

[38] Bolz J, ParaGym. Partizipative Entwicklung einer personalisierbaren Trainings-App für Menschen mit Paraplegie – lähmungsspezifische Anforderungen an den Übungskatalog. In: 35. Jahrestagung der Deutschsprachigen Medizinischen Gesellschaft für Paraplegiologie. Spätfolgen der Querschnittlähmung: Prävention und Therapie. Bad Wildungen, Germany; 2022. p. 27.

[39] Bolz J, Nickel K, Bölecke L, Löscher A. Partizipative Entwicklung einer sensorgestützten Trainings-App für Menschen mit Paraplegie. Orthopädie Technik. 2022;73:42–8. https://360-ot.de/partizipative-entwicklung-einer-sensorgestuetzten-trainings-app-fuer-menschen-mit-paraplegie/.

[40] Baehr LA, Hiremath SV, Bruneau M, Chiarello LA, Kaimal G, Newton R, et al. Effect of Tele-exercise to Promote Empowered Movement for Individuals With Spinal Cord Injury (TEEMS) Program on Physical Activity Determinants and Behavior: A Mixed Methods Assessment. Arch Phys Med Rehabil. 2024;105:101–11. 10.1016/j.apmr.2023.08.019. 37678447 10.1016/j.apmr.2023.08.019

[41] Wingood M, Gell NM. Physical Therapist Engagement With a Home Exercise Prescription Platform: A Mixed Methods Study. Arch Phys Med Rehabil. 2025. 10.1016/j.apmr.2025.06.005. ;:S0003999325007580. 40544928 10.1016/j.apmr.2025.06.005

[42] Palinkas LA, Aarons GA, Horwitz S, Chamberlain P, Hurlburt M, Landsverk J. Mixed Method Designs in Implementation Research. Adm Policy Ment Health. 2011;38:44–53. 10.1007/s10488-010-0314-z. 20967495 10.1007/s10488-010-0314-z PMC3025112

[43] Creswell JW, Creswell JD. Research design: qualitative, quantitative, and mixed methods approaches. Fifth edition. Los Angeles London New Delhi Singapore Washington DC Melbourne: SAGE; 2018.

[44] Fetters MD, Curry LA, Creswell JW. Achieving Integration in Mixed Methods Designs—Principles and Practices. Health Serv Res. 2013;48:2134–56. 10.1111/1475-6773.12117. 24279835 10.1111/1475-6773.12117 PMC4097839

[45] Kuckartz U, Rädiker S. Analyzing Qualitative Data with MAXQDA: Text, Audio, and Video. Cham: Springer International Publishing; 2019. 10.1007/978-3-030-15671-8.

[46] Bolz J, Perret C, Predel H-G. Development of an intelligent exercise app prototype for individuals with paraplegia: An online questionnaire-based requirement analysis. In: ISCoS 2023 Oral & rapid fire presentations abstract book. Edinburgh; 2023. p. 81; Paper number 397.

[47] World Medical Association (WMA). WMA declaration of helsinki – ethical principles for medical research involving human participants. 2024. https://www.wma.net/policies-post/wma-declaration-of-helsinki/. Accessed 20 Apr 2026.

[48] Tong A, Sainsbury P, Craig J. Consolidated criteria for reporting qualitative research (COREQ): a 32-item checklist for interviews and focus groups. Int J Qual Health Care. 2007;19:349–57. 10.1093/intqhc/mzm042. 17872937 10.1093/intqhc/mzm042

[49] O’Brien BC, Harris IB, Beckman TJ, Reed DA, Cook DA. Standards for Reporting Qualitative Research: A Synthesis of Recommendations. Acad Med. 2014;89:1245–51. 10.1097/ACM.0000000000000388. 24979285 10.1097/ACM.0000000000000388

[50] O’Cathain A, Murphy E, Nicholl J. The Quality of Mixed Methods Studies in Health Services Research. J Health Serv Res Policy. 2008;13:92–8. 10.1258/jhsrp.2007.007074. 18416914 10.1258/jhsrp.2007.007074

[51] Warburton DER, Jamnik VK, Bredin SSD, Gledhill N. The Physical Activity Readiness Questionnaire for Everyone (PAR-Q+) and Electronic Physical Activity Readiness Medical Examination (ePARmed-X+). Health Fit J Can. 2011;3–17. 10.14288/HFJC.V4I2.103.

[52] Martin Ginis KA, van der Scheer JW, Latimer-Cheung AE, Barrow A, Bourne C, Carruthers P, et al. Evidence-based scientific exercise guidelines for adults with spinal cord injury: an update and a new guideline. Spinal Cord. 2018;56:308–21. 10.1038/s41393-017-0017-3. 29070812 10.1038/s41393-017-0017-3

[53] Faulkner L. Beyond the five-user assumption: Benefits of increased sample sizes in usability testing. Behav Res Methods Instrum Comput. 2003;35:379–83. 10.3758/BF03195514. 14587545 10.3758/bf03195514

[54] Billingham SA, Whitehead AL, Julious SA. An audit of sample sizes for pilot and feasibility trials being undertaken in the United Kingdom registered in the United Kingdom Clinical Research Network database. BMC Med Res Methodol. 2013;13:104. 10.1186/1471-2288-13-104. 23961782 10.1186/1471-2288-13-104 PMC3765378

[55] Viechtbauer W, Smits L, Kotz D, Budé L, Spigt M, Serroyen J, et al. A simple formula for the calculation of sample size in pilot studies. J Clin Epidemiol. 2015;68:1375–9. 10.1016/j.jclinepi.2015.04.014. 26146089 10.1016/j.jclinepi.2015.04.014

[56] Bentley CL, Powell L, Potter S, Parker J, Mountain GA, Bartlett YK, et al. The Use of a Smartphone App and an Activity Tracker to Promote Physical Activity in the Management of Chronic Obstructive Pulmonary Disease: Randomized Controlled Feasibility Study. JMIR Mhealth Uhealth. 2020;8:e16203. 10.2196/16203. 32490838 10.2196/16203 PMC7301262

[57] Bolz J. Konzept zur Entwicklung einer „Fitness-App für Menschen mit Paraplegie und zur Steigerung der Gesundheitskompetenz im Rahmen des FIT-IN3-Projekts. Masterarbeit. Deutsche Sporthochschule Köln; 2021.

[58] Chavez A. What Are Semi-Structured Interviews? 2026. https://www.maxqda.com/research-guides/semi-structured-interviews. Accessed 22 Apr 2026.

[59] Barisch-Fritz B, Bezold J, Scharpf A, Trautwein S, Krell-Roesch J, Woll A. ICT-Based Individualized Training of Institutionalized Individuals With Dementia. Evaluation of Usability and Trends Toward the Effectiveness of the InCoPE-App. Front Physiol. 2022;13:921105. 10.3389/fphys.2022.921105. 35874545 10.3389/fphys.2022.921105 PMC9304760

[60] Burkow TM, Vognild LK, Johnsen E, Bratvold A, Risberg MJ. Promoting exercise training and physical activity in daily life: a feasibility study of a virtual group intervention for behaviour change in COPD. BMC Med Inf Decis Mak. 2018;18:136. 10.1186/s12911-018-0721-8. 10.1186/s12911-018-0721-8 PMC6299608 30563507

[61] Buss VH, Varnfield M, Harris M, Barr M. A Mobile App for Prevention of Cardiovascular Disease and Type 2 Diabetes Mellitus: Development and Usability Study. JMIR Hum Factors. 2022;9:e35065. 10.2196/35065. 35536603 10.2196/35065 PMC9131155

[62] Daly RM, Gianoudis J, Hall T, Mundell NL, Maddison R, Feasibility. Usability, and Enjoyment of a Home-Based Exercise Program Delivered via an Exercise App for Musculoskeletal Health in Community-Dwelling Older Adults: Short-term Prospective Pilot Study. JMIR Mhealth Uhealth. 2021;9:e21094. 10.2196/21094. 33439147 10.2196/21094 PMC7840282

[63] Jaffar A, Muhammad N, Mohd Sidik S, Admodisastro N, Abdul Manaf R, Foo C, et al. Feasibility and Usability of Kegel Exercise Pregnancy Training App (KEPT App) among Pregnant Women with Urinary Incontinence. IJERPH. 2022;19:3574. 10.3390/ijerph19063574. 35329262 10.3390/ijerph19063574 PMC8955097

[64] Juengst SB, McShan E, Conley M, Luu I, Driver S. Feasibility and Pilot Testing of Mobile Health Apps to Supplement 2 Healthy Lifestyle Interventions in Chronic Traumatic Brain Injury. J Head Trauma Rehabilitation. 2022;37:162–70. 10.1097/HTR.0000000000000769. 10.1097/HTR.0000000000000769 35293364

[65] Landers MR, Ellis TD. A Mobile App Specifically Designed to Facilitate Exercise in Parkinson Disease: Single-Cohort Pilot Study on Feasibility, Safety, and Signal of Efficacy. JMIR Mhealth Uhealth. 2020;8:e18985. 10.2196/18985. 33016887 10.2196/18985 PMC7573700

[66] Loh KP, Sanapala C, Watson EE, Jensen-Battaglia M, Janelsins MC, Klepin HD, et al. A single-arm pilot study of a mobile health exercise intervention (GO-EXCAP) in older patients with myeloid neoplasms. Blood Adv. 2022;6:3850–60. 10.1182/bloodadvances.2022007056. 35320340 10.1182/bloodadvances.2022007056 PMC9278283

[67] Nicholas JC, Ntoumanis N, Smith BJ, Quested E, Stamatakis E, Thøgersen-Ntoumani C. Development and feasibility of a mobile phone application designed to support physically inactive employees to increase walking. BMC Med Inf Decis Mak. 2021;21:23. 10.1186/s12911-021-01391-3. 10.1186/s12911-021-01391-3 PMC7819207 33478495

[68] Nicolaidou I, Aristeidis L, Lambrinos L. A gamified app for supporting undergraduate students’ mental health: A feasibility and usability study. Digit Health. 2022;8:205520762211090. 10.1177/20552076221109059. 10.1177/20552076221109059 PMC9228636 35756831

[69] Signorelli GR, Monteiro-Guerra F, Rivera-Romero O, Núñez-Benjumea FJ, Fernández-Luque L. Breast Cancer Physical Activity Mobile Intervention: Early Findings From a User Experience and Acceptability Mixed Methods Study. JMIR Form Res. 2022;6:e32354. 10.2196/32354. 35731554 10.2196/32354 PMC9260535

[70] Stephenson A, Garcia-Constantino M, Murphy MH, McDonough SM, Nugent CD, Mair JL. The Worktivity mHealth intervention to reduce sedentary behaviour in the workplace: a feasibility cluster randomised controlled pilot study. BMC Public Health. 2021;21:1416. 10.1186/s12889-021-11473-6. 34275463 10.1186/s12889-021-11473-6 PMC8286585

[71] Tong HL, Quiroz JC, Kocaballi AB, Ijaz K, Coiera E, Chow CK, et al. A personalized mobile app for physical activity: An experimental mixed-methods study. Digit HEALTH. 2022;8:205520762211150. 10.1177/20552076221115017. 10.1177/20552076221115017 PMC9309778 35898287

[72] Tonga E, Williamson E, Srikesavan C, Özen T, Sarıtaş F, Lamb SE. A hand exercise mobile app for people with rheumatoid arthritis in Turkey: design, development and usability study. Rheumatol Int. 2021;41:1151–60. 10.1007/s00296-021-04860-0. 33870452 10.1007/s00296-021-04860-0

[73] Bowen DJ, Kreuter M, Spring B, Cofta-Woerpel L, Linnan L, Weiner D, et al. How We Design Feasibility Studies. Am J Prev Med. 2009;36:452–7. 10.1016/j.amepre.2009.02.002. 19362699 10.1016/j.amepre.2009.02.002 PMC2859314

[74] Borg G. Borg’s Perceived exertion and pain scales. Champaign, IL: Human Kinetics; 1998.

[75] Laird B, Puzia M, Larkey L, Ehlers D, Huberty J. A Mobile App for Stress Management in Middle-Aged Men and Women (Calm): Feasibility Randomized Controlled Trial. JMIR Form Res. 2022;6:e30294. 10.2196/30294. 34989677 10.2196/30294 PMC9132144

[76] Brooke J. SUS: A quick and dirty usability scale. In: Jordan PW, Thomas B, Weerdmeester BA, McClelland IL, editors. Usability evaluation in industry. London; Bristol, Pa: Taylor & Francis; 1996. pp. 189–94.

[77] Bangor A, Kortum PT, Miller JT. An Empirical Evaluation of the System Usability Scale. Int J Hum Comput Interact. 2008;24:574–94. 10.1080/10447310802205776.

[78] Rampersaud YR, Anderson PA, Dimar JR, Fisher CG. __. Spinal Adverse Events Severity System, version 2 (SAVES-V2): inter- and intraobserver reliability assessment. J Neurosurg Spine. 2016;25:256–63. 10.3171/2016.1.SPINE14808. 27058499 10.3171/2016.1.SPINE14808

[79] Dresing T, Pehl T, Praxisbuch Interview. Transkription & Analyse: Anleitungen und Regelsysteme für qualitativ Forschende. 8. Auflage. Marburg: Eigenverlag; 2018.

[80] Kuckartz U, Rädiker S. Qualitative Inhaltsanalyse: Methoden, Praxis, Computerunterstützung: Grundlagentexte Methoden. 5. Auflage. Weinheim Basel: Beltz Juventa; 2022.

[81] Widerström-Noga E, Anderson KD, Perez S, Hunter JP, Martinez-Arizala A, Adcock JP, et al. Living With Chronic Pain After Spinal Cord Injury: A Mixed-Methods Study. Arch Phys Med Rehabil. 2017;98:856–65. 10.1016/j.apmr.2016.10.018. 27894730 10.1016/j.apmr.2016.10.018

[82] Kuckartz U, Rädiker S. Qualitative content analysis: methods, practice and software. 2nd edition. Los Angeles ; London ; New Delhi ; Singapore ; Washington DC ; Melbourne: SAGE; 2023.

[83] Froehlich-Grobe K, Lee J, Ochoa C, Lopez A, Sarker E, Driver S, et al. Effectiveness and feasibility of the workout on wheels internet intervention (WOWii) for individuals with spinal cord injury: a randomized controlled trial. Spinal Cord. 2022;60:862–74. 10.1038/s41393-022-00787-w. 35474116 10.1038/s41393-022-00787-w PMC9041282

[84] Wilroy JD, Lai B, Davlyatov G, Mehta T, Thirumalai M, Rimmer JH. Correlates of adherence in a home-based, self-managed exercise program tailored to wheelchair users with spinal cord injury. Spinal Cord. 2021;59:55–62. 10.1038/s41393-020-0497-4. 32541883 10.1038/s41393-020-0497-4 PMC7962257

[85] van den Akker LE, Holla JFM, Dadema T, Visser B, Valent LJ, de Groot S, et al. Determinants of physical activity in wheelchair users with spinal cord injury or lower limb amputation: perspectives of rehabilitation professionals and wheelchair users. Disabil Rehabil. 2020;42:1934–41. 10.1080/09638288.2019.1577503. 30924706 10.1080/09638288.2019.1577503

[86] Peng W, Kanthawala S, Yuan S, Hussain SA. A qualitative study of user perceptions of mobile health apps. BMC Public Health. 2016;16:1158. 10.1186/s12889-016-3808-0. 27842533 10.1186/s12889-016-3808-0 PMC5109835

[87] Pancer M, Manganaro M, Pace I, Marion P, Gagnon DH, Laramée M-T, et al. A Web-Based Physical Activity Portal for Individuals Living With a Spinal Cord Injury: Qualitative Study. JMIR Form Res. 2019;3:e12507. 10.2196/12507. 31350835 10.2196/12507 PMC6688442

[88] Stephens C, Neil R, Smith P. The perceived benefits and barriers of sport in spinal cord injured individuals: a qualitative study. Disabil Rehabil. 2012;34:2061–70. 10.3109/09638288.2012.669020. 22494335 10.3109/09638288.2012.669020

[89] Levins SM, Redenbach DM, Dyck I. Individual and Societal Influences on Participation in Physical Activity Following Spinal Cord Injury: A Qualitative Study. Phys Ther. 2004;84:496–509. 10.1093/ptj/84.6.496. 15161416

[90] Kehn M, Kroll T. Staying physically active after spinal cord injury: a qualitative exploration of barriers and facilitators to exercise participation. BMC Public Health. 2009;9:168. 10.1186/1471-2458-9-168. 19486521 10.1186/1471-2458-9-168 PMC2694784

[91] Riebe D, Franklin BA, Thompson PD, Garber CE, Whitfield GP, Magal M, et al. Updating ACSM’s Recommendations for Exercise Preparticipation Health Screening. Med Sci Sports Exerc. 2015;47:2473–9. 10.1249/MSS.0000000000000664. 26473759 10.1249/MSS.0000000000000664

