# Supplementary material for: Automatically tailored exercise app training is feasible, usable, and safe for people with paraplegia: a parallel mixed methods pilot study
Source: BMC Sports Sci Med Rehabil. 2026 Jun 12;18:273. doi: 10.1186/s13102-026-01801-x (PMC13261973; doi:10.1186/s13102-026-01801-x)
Supplement: Supplementary file 1 — Additional file 1: PDF; Reporting guidelines checklists: COREQ, SRQR; GRAMMS. [file 13102_2026_1801_MOESM1_ESM.pdf]

The Consolidated Criteria for Reporting Qualitative Research (COREQ) checklist

Tong A, Sainsbury P, Craig J. Consolidated criteria for reporting qualitative research (COREQ): a 32-item checklist for interviews and focus groups. *International Journal for Quality in Health Care*. 2007. Volume 19, Number 6: pp. 349 – 357

No. Item	Guide questions/description	Reported in Section*
**Domain 1: Research team and reflexivity**		
Personal Characteristics		
1. Interviewer/facilitator	Which author/s conducted the interview or focus group?	Outcomes
2. Credentials	What were the researcher's credentials? E.g. PhD, MD	Title Page; Outcomes
3. Occupation	What was their occupation at the time of the study?	Outcomes; Qualitative Analyses
4. Gender	Was the researcher male or female?	Outcomes; Qualitative Analyses
5. Experience and training	What experience or training did the researcher have?	Outcomes; Qualitative Analyses
Relationship with participants		
6. Relationship established	Was a relationship established prior to study commencement?	Qualitative analysis
7. Participant knowledge of the interviewer	What did the participants know about the researcher? e.g. personal goals, reasons for doing the research	Outcomes Limitations
8. Interviewer characteristics	What characteristics were reported about the interviewer/facilitator? e.g. Bias, assumptions, reasons and interests in the research topic	Outcomes; Qualitative Analyses
**Domain 2: Study design**		
Theoretical framework		
9. Methodological orientation and Theory	What methodological orientation was stated to underpin the study? e.g. grounded theory, discourse analysis, ethnography, phenomenology, content analysis	Study Design; Qualitative Analyses; Additional File 3
Participant selection		

10. Sampling	How were participants selected? e.g. purposive, convenience, consecutive, snowball	Participants (Methods)
11. Method of approach	How were participants approached? e.g. face-to-face, telephone, mail, email	Participants (Methods)
12. Sample size	How many participants were in the study?	Participants (Results) Table 1; Figure 3
13. Non-participation	How many people refused to participate or dropped out? Reasons?	Adherence and Retention Rates (Results), Figure 3
Setting		
14. Setting of data collection	Where was the data collected? e.g. home, clinic, workplace	Study Design
15. Presence of non-participants	Was anyone else present besides the participants and researchers?	Outcomes
16. Description of sample	What are the important characteristics of the sample? e.g. demographic data, date	Participants (Results) Table 1
Data collection		
17. Interview guide	Were questions, prompts, guides provided by the authors? Was it pilot tested?	Outcomes;<br>Additional File 3
18. Repeat interviews	Were repeat interviews carried out? If yes, how many?	Study; Figure 2
19. Audio/visual recording	Did the research use audio or visual recording to collect the data?	Outcomes
20. Field notes	Were field notes made during and/or after the interview or focus group?	Outcomes
21. Duration	What was the duration of the interviews or focus group?	Results
22. Data saturation	Was data saturation discussed?	Outcomes
23. Transcripts returned	Were transcripts returned to participants for comment and/or correction?	Qualitative Analyses; Limitations
**Domain 3: analysis and findings**		
Data analysis		

24. Number of data coders	How many data coders coded the data?	Qualitative Analyses
25. Description of the coding tree	Did authors provide a description of the coding tree?	Additional file 3
26. Derivation of themes	Were themes identified in advance or derived from the data?	Qualitative Analyses
27. Software	What software, if applicable, was used to manage the data?	Qualitative Analyses; Quantitative Analyses
28. Participant checking	Did participants provide feedback on the findings?	Qualitative Analyses
Reporting		
29. Quotations presented	Were participant quotations presented to illustrate the themes/findings? Was each quotation identified? e.g. participant number	Qualitative Analyses;<br>Feasibility (Results);<br>Usability (Results);<br>Safety (Results);<br>Additional File 4
30. Data and findings consistent	Was there consistency between the data presented and the findings?	Feasibility (Discussion);<br>Usability (Discussion);<br>Safety (Discussion)
31. Clarity of major themes	Were major themes clearly presented in the findings?	Figure 4;<br>Feasibility (Results);<br>Usability (Results);<br>Safety (Results);<br>Feasibility (Discussion);<br>Usability (Discussion);<br>Safety (Discussion);<br>Implications;<br>Additional File 4
32. Clarity of minor themes	Is there a description of diverse cases or discussion of minor themes?	Figure 4;<br>Feasibility (Results);<br>Usability (Results);<br>Safety (Results);<br>Feasibility (Discussion);<br>Usability (Discussion);<br>Safety (Discussion);<br>Implications;<br>Additional File 4
*Since the page numbers differ between the submitted and published manuscripts, page numbers are not included here; instead, the relevant section is indicated.		

Standards for Reporting Qualitative Research (SRQR)

Bridget ‘C. O’Brien, Ilene B. Harris, Thomas J. Beckman, Darcy A. Reed & David A. Cook. Standards for Reporting Qualitative Research: A Synthesis of Recommendations. *Academic Medicine*. 2014. Volume 89, Number 9: pp. 1245 – 1251

No.	Topic	Item	Reported in Section*
Title and abstract			
S1	Title	Concise description of the nature and topic of the study Identifying the study as qualitative or indicating the approach (e.g., ethnography, grounded theory) or data collection methods (e.g., interview, focus group) is recommended	Title Page
S2	Abstract	Summary of key elements of the study using the abstract format of the intended publication; typically includes background, purpose, methods, results, and conclusions	Abstract
Introduction			
S3	Problem formulation	Description and significance of the problem/phenomenon studied; review of relevant theory and empirical work; problem statement	Introduction
S4	Purpose or research question	Purpose of the study and specific objectives or questions	Introduction
Methods			
S5	Qualitative approach and research paradigm	Qualitative approach (e.g., ethnography, grounded theory, case study, phenomenology, narrative research) and guiding theory if appropriate; identifying the research paradigm (e.g., postpositivist, constructivist/ interpretivist) is also recommended; rationale [a]	Study Design; Qualitative Analyses
S6	Researcher characteristics and reflexivity	Researchers’ characteristics that may influence the research, including personal attributes, qualifications/experience, relationship with participants, assumptions, and/or presuppositions; potential or actual interaction between researchers’ characteristics and the research questions, approach, methods, results, and/or transferability	Outcomes; Qualitative Analyses
S7	Context	Setting/site and salient contextual factors; rationale [a]	Study Design; Outcomes

S8	Sampling strategy	How and why research participants, documents, or events were selected; criteria for deciding when no further sampling was necessary (e.g., sampling saturation); rationale [a]	Participants (Methods)
S9	Ethical issues pertaining to human subjects	Documentation of approval by an appropriate ethics review board and participant consent, or explanation for lack thereof; other confidentiality and data security issues	Study Design
S10	Data collection methods	Types of data collected; details of data collection procedures including (as appropriate) start and stop dates of data collection and analysis, iterative process, triangulation of sources/methods, and modification of procedures in response to evolving study findings; rationale [a]	Study Design; Outcomes; Quantitative Analyses; Qualitative Analyses; Mixed Methods Integration
S11	Data collection instruments and technologies	Description of instruments (e.g., interview guides, questionnaires) and devices (e.g., audio recorders) used for data collection; if/how the instrument(s) changed over the course of the study	Outcomes; Additional File 4
S12	Units of study	Number and relevant characteristics of participants, documents, or events included in the study; level of participation (could be reported in results)	Participants (Results)
S13	Data processing	Methods for processing data prior to and during analysis, including transcription, data entry, data management and security, verification of data integrity, data coding, and anonymization/deidentification of excerpts	Quantitative Analyses; Qualitative Analyses; Additional File 4
S14	Data analysis	Process by which inferences, themes, etc., were identified and developed, including the researchers involved in data analysis; usually references a specific paradigm or approach; rationale [a]	Qualitative analyses; Additional File 4
S15	Techniques to enhance trustworthiness	Techniques to enhance trustworthiness and credibility of data analysis (e.g., member checking, audit trail, triangulation); rationale [a]	Qualitative analyses; Limitations
Results/findings			
S16	Synthesis and interpretation	Main findings (e.g., interpretations, inferences, and themes); might include development of a theory or model, or integration with prior research or theory	Figure 4; Feasibility (Results); Usability (Results); Safety (Results); Feasibility (Discussion); Usability (Discussion);

			Safety (Discussion); Implications; Additional File 4
S17	Links to empirical data	Evidence (e.g., quotes, field notes, text excerpts, photographs) to substantiate analytic findings	Feasibility (Results); Usability (Results); Safety (Results); Additional File 4
Discussion			
S18	Integration with prior work, implications, transferability, and contribution(s) to the field	Short summary of main findings; explanation of how findings and conclusions connect to, support, elaborate on, or challenge conclusions of earlier scholarship; discussion of scope of application/ generalizability; identification of unique contribution(s) to scholarship in a discipline or field	Feasibility (Discussion); Usability (Discussion); Safety (Discussion); Implications
S19	Limitations	Trustworthiness and limitations of findings	Limitations
Other			
S20	Conflicts of interest	Potential sources of influence or perceived influence on study conduct and conclusions; how these were managed	Acknowledgments
S21	Funding	Sources of funding and other support; role of funders in data collection, interpretation, and reporting	Acknowledgments

*Since the page numbers differ between the submitted and published manuscripts, page numbers are not included here; instead, the relevant section is indicated.

[a] The rationale should briefly discuss the justification for choosing that theory, approach, method, or technique rather than other options available, the assumptions and limitations implicit in those choices, and how those choices influence study conclusions and transferability. As appropriate, the rationale for several items might be discussed together.

Good Reporting of A Mixed Methods Study (GRAMMS)

Alicia O'Cathain, Elisabeth Murphy, & Jon Nicholi. The quality of mixed methods studies in health services research. *Journal of Health Services Research & Policy*. 2008. Volume 13, Number 2: pp: 92-98.

No.	Item	Reported in Section*
1	Describe the justification for using a mixed methods approach to the research question	Study Design and Intervention
2	Describe the design in terms of the purpose, priority and sequence of methods	Study Design and Intervention
3	Describe each method in terms of sampling, data collection and analysis	Participants;<br>Outcomes;<br>Quantitative Analyses;<br>Qualitative Analyses
4	Describe where integration has occurred, how it has occurred and who has participated in it.	Mixed Methods Integration
5	Describe any limitation of one method associated with the present of the other method	Limitations
6	Describe any insights gained from mixing or integrating methods	Feasibility (Discussion);<br>Usability (Discussion);<br>Safety (Discussion)
*Since the page numbers differ between the submitted and published manuscripts, page numbers are not included here; instead, the relevant section is indicated.		
