# Supplementary material for: Automatically tailored exercise app training is feasible, usable, and safe for people with paraplegia: a parallel mixed methods pilot study
Source: BMC Sports Sci Med Rehabil. 2026 Jun 12;18:273. doi: 10.1186/s13102-026-01801-x (PMC13261973; doi:10.1186/s13102-026-01801-x)
Supplement: Supplementary file 2 — Additional file 2: PDF; Exercise diary and sample workout. [file 13102_2026_1801_MOESM2_ESM.pdf]

# Exercise Diary

Dear study participant,

With this study, we aim to examine both the functionality of the app and possible health effects of training using the app. To this end, we will collect data on performance, handgrip strength, independence in everyday life, and health-related quality of life at the beginning and end of the intervention period. Engaging in physical activity during the intervention period, for example as part of therapy or privately with a handbike, may impact test results. We therefore ask you to document all physical activities (for the training group, including sessions with the ParaGym app), as well as the respective duration and intensity using this exercise diary. This way those activities can be taken into account when interpreting the results.

Instructions for filling out the form:

- Please write your participant ID on each sheet of the exercise diary. If a sheet falls out, we will still be able to identify it as yours.
- In the first column, please write the date on which you completed the exercise session.
- Then note the type of sporting activity in the second column.
- On the right, you will find the Borg scale for assessing your subjective perception of exertion. Please rate your session using this scale and enter the corresponding numerical value in the third column.
- Please enter the total duration of your session in the fourth column.
- The fifth column is for any other comments. This applies in particular to participants who have trained with the ParaGym app. Please enter anything you noticed during training with the app, e.g., regarding the functionality of the app, the suitability of the exercises, or safety.

Table 1. Subjective perception of exertion according to the Borg RPE scale

| | |
|---|---|
| 6 | No exertion at all |
| 7 | Extremely light |
| 8 | |
| 9 | Very light |
| 10 | |
| 11 | Light |
| 12 | |
| 13 | Somewhat hard |
| 14 | |
| 15 | Hard (heavy) |
| 16 | |
| 17 | Very hard |
| 18 | |
| 19 | Extremely hard |
| 20 | Maximal exertion |

| My exercise diary | | | | | Participant ID |
|---|---|---|---|---|---|
| Date | My activities (e.g., therapy, handbike, ParaGym, etc.) | Intensity (Borg Scale) | Duration (min) | Comments | |
| | | | | | |
| | | | | | |
| | | | | | |
| | | | | | |
| | | | | | |

## Sample Workout

**This sample workout was created solely to demonstrate the variety and scalability of the exercises included in the ParaGym app. It was not developed based on findings from exercise or sports science, and it does not claim therapeutic value.**

| **Warm-up** | |
|---|---|
| Cervical Spine Mobilization<br>Reach-up stretch<br>Scapula Mobilization<br>Hand Rotation<br>Sitting Jacks | |
| **Part 1 (incomplete lesion)** | **Part 1 (complete lesion)** |
| Banded Row*<br>Synchronous Chairwalks Forwards<br>Reverse Dips from Wheelchair<br>Banded Shoulder External Rotation<br>Banded Seatbelt | Banded Row*<br>Half Package in Sitting<br>Reverse Dips in Wheelchair<br>Banded Shoulder External Rotation<br>Chop & Lift |
| **Part 2 (incomplete lesion)** | **Part 2 (complete lesion)** |
| Burpees*<br>Banded Reverse Butterfly<br>Banded Diagonal Crunches<br>High to Low Wheely<br>Banded Antirotation | Burpees*<br>Bodyweight Reverse Butterfly<br>Wheeltaps<br>Line Hoppings<br>Spinal Rotation in Sitting |
| **Cool-down** | |
| Pectoralis Stretch<br>Lower Arm Stretch<br>Triceps Stretch<br>Single Leg Seated Hamstring Stretch<br>Hip internal Rotator Stretch in Sitting | |

* Two alternatives were included in the exercise video
