# Supplementary material for: Automatically tailored exercise app training is feasible, usable, and safe for people with paraplegia: a parallel mixed methods pilot study
Source: BMC Sports Sci Med Rehabil. 2026 Jun 12;18:273. doi: 10.1186/s13102-026-01801-x (PMC13261973; doi:10.1186/s13102-026-01801-x)
Supplement: Supplementary file 3 — Additional file 3: PDF; Interview analysis material: Interview Guide, Transcription Rules, Codebook, Coding Guideline. [file 13102_2026_1801_MOESM3_ESM.pdf]

Interview Guide

Original (German) – English translation see below

Topic	Questions	Prompts
Opening<br><br>Overall Experience / Satisfaction	**Wie viel Erfahrung hast du mit der Nutzung von Gesundheits- oder Sport-Apps?**	- Wofür genutzt?<br>- Was hat gefallen?<br>- Was nicht?<br>- Funktionen vermisst?
	**Was hat dir am Training mit dem Prototyp der ParaGym-App gut gefallen?**	Individualisierung/Zuschneidung<br>Equipment<br>Übungsauswahl<br>Aufbau Trainingseinheiten<br>Nutzungsfreundlichkeit<br>Übungsvideos<br>Übungsbeschreibungen
	**Was hat dir am Training mit dem Prototyp der ParaGym-App weniger gut gefallen?**<br>- Was sollte anders gemacht/gelöst werden?<br>- Hast du Ideen, wie das Problem gelöst werden könnte?	Individualisierung/Zuschneidung<br>Equipment<br>Technische Probleme<br>Übungsauswahl<br>Aufbau Trainingseinheiten<br>Nutzungsfreundlichkeit<br>Übungsvideos<br>Übungsbeschreibungen
Ease of use	**Wie würdest du die Benutzungsfreundlichkeit der App bewerten?**<br>- Was ist dir bei der Benutzung leicht gefallen?<br>- Was ist dir schwer gefallen?	Definition Benutzungsfreundlichkeit<br>Registrierung<br>Übersichtlichkeit<br>Design<br>Intuitive Bedienung<br>Umgang mit der App

App functionality	**Wie findest du die Gestaltung der Trainingseinheiten?**	Spaß<br>Vielfältigkeit/Abwechslungsreichtum/Auswahl<br>Anspruch der Übungen<br>Dauer einer Einheit<br>Intensität<br>Equipment
	**Wie hast du die Individualisierung durch den Algorithmus wahrgenommen?**	Anpassung bemerkt?<br>Anpassung angemessen?<br>Zufriedenheit mit Anpassung<br>Equipment<br>Sperrung von Übungen
Safety	**Hast du dich während des Trainings sicher gefühlt?**<br>- Warum?<br>- Warum nicht?<br>- Was muss sich ändern, damit du dich sicherer fühlst?	Befinden beim Training<br>Hilfestellung durch andere Person
	**Hast du während des Interventionszeitraumes Kontakt zu Therapeut:innen oder Ärzt:innen in Zusammenhang mit der ParaGym-App aufgenommen?**<br>- Wenn ja, warum?	
Technical Functionality	**Gab es technische Probleme bei der Benutzung der App?**<br>- Wenn ja, welche?<br>- Hast du diese lösen können und wenn ja, wie?	Laden der Videos<br>Individualisierung<br>Beenden der Workouts
Communication	**Wie bewertest du die Kommunikation zu dem Projektteam?**	Erreichbarkeit<br>Häufigkeit des Kontakts<br>Qualität der Informationen
Perceived Effects/ Usefulness	**Hast du das Gefühl, dass sich durch das Training mit ParaGym etwas verändert hat?**	Leistungsfähigkeit<br>Körperliche Gesundheit<br>Psychische Gesundheit

	**Hat dir die App geholfen, im Alltag körperlich aktiver zu sein?**	Energie<br>Motivation<br>Partizipation<br>Mobilität
Adherence/ Motivation	**Wie regelmäßig hast du mit dem Prototyp der App über die sechs Wochen trainiert?**<br>- Was hat dich zum Training motiviert?<br>- Was hat dich am Training gehindert?	durchschnittliche Anzahl der Einheiten pro Woche<br>Förderfaktoren<br>Barrieren
	**Würdest auch über die Studie hinaus mit dem Prototyp der ParaGym-App oder einer marktreifen Version dieser trainieren?**<br>- Welche Eigenschaften oder Funktionen müsste die App haben, damit du gerne weiter damit trainierst?	Langzeitmotivation
Ending/ Recommendation	**Würdest du das Training mit ParaGym weiterempfehlen?**<br>- Warum?<br>- Warum nicht?<br>- Oder das Training mit einer weiterentwickelten App	
	**Gibt es etwas, was du uns sonst noch mitgeben möchtest?**	

English translation

**Topic**	**Questions**	**Prompts**
Opening<br><br>Overall Experience / Satisfaction	**How much experience do you have with using health or sports apps?**	- What purpose?<br>- What did you like about it?<br>- Disliked?<br>- Functions/features missed?

	**What did you like about training with the prototype of the ParaGym app?**	Individualization<br>Equipment<br>Exercise selection<br>Structure training sessions<br>Ease of use<br>Videos<br>Descriptions
	**What did you like least about training with the prototype of the ParaGym app?**<br>- What should be done differently/resolved?<br>- Do you have any ideas on how this issue could be resolved?	Individualization<br>Equipment<br>Technical issues<br>Exercise selection<br>Structure training sessions<br>Ease of use<br>Videos<br>Descriptions
Ease of use	**How would you rate the app's user-friendliness?**<br>- What did you find easy to use?<br>- What did you find difficult?	Definition usability<br>registration<br>Layout<br>Design<br>Intuitive handling<br>Using the app
App functionality	**What do you think of the training sessions?**	Fun<br>Variety / selection<br>Exercise difficulty<br>Duration<br>Intensity<br>Equipment
	**How did you perceive the individualization through the algorithm?**	Noticed individualization?<br>adaption adequate?<br>Satisfaction<br>Equipment<br>Blocking of exercises

Safety	**Did you feel safe during the training?**<br>- What made you feel safe?<br>- What didn‘t?<br>- What needs to change for you to feel safer?	Condition during training<br>Assistance from another person
	**Did you contact therapists or doctors in connection with the ParaGym app during the intervention period?**<br>- If so, why?	
Technical Functionality	**Did you encounter any technical problems when using the app?**<br>- If so, which?<br>- Were you able to solve them, and if so, how?	Loading videos<br>Customization<br>Ending workouts
Communication	**How would you rate communication with the project team?**	Availability<br>Frequency of contact<br>Quality of information
Perceived Effects/ Usefulness	**Do you feel that training with ParaGym has made a difference?**	Performance capacity<br>Physical health<br>Mental health
	**Has the app helped you to be more physically active in your everyday life?**	Energy<br>Motivation<br>Participation<br>Mobility
Adherence/ Motivation	**How regularly did you train with the prototype app over the six weeks?**<br>- What motivated you to train?<br>- What prevented you from training?	Average number of sessions per week<br>Promoting factors<br>Barriers
	**Would you continue training with the ParaGym app prototype or a market-ready version of it even after the study?**<br>- What features or functions would the app need to have for you to want to continue training with it?	Long-term motivation

Ending/<br>Recommendation	**Would you recommend the app to your peers?**<br>- Why?<br>- Why not?<br>- Would you recommend the app if it was further developed?	
	**Is there anything else, that you would like to share with us?**	

Transcription Rules

	Original (German)	English
	**Inhaltlich-semantische Transkription und Hinweise zur einheitlichen Schreibweise**	**Content-semantic transcription and instruction on consistent spelling**
1	Es wird wörtlich transkribiert, also nicht lautsprachlich oder zusammenfassend.	Transcription is literal, i.e., not phonetic or summarized
2	Wortverschleifungen werden an das Schriftdeutsch angenähert. „So ‘n Buch“ wird zu „so ein Buch“ und „hamma“ wird zu „haben wir“. Die Satzform wird beibehalten, auch wenn sie syntaktische Fehler beinhaltet, z.B.: „Bin ich nach Kaufhaus gegangen.“	Word slurring is approximated to written German. “So 'n Buch” becomes “so ein Buch” and ‘hamma’ becomes “haben wir.” The sentence structure is retained, even if it contains syntactical errors, e.g.: “Bin ich nach Kaufhaus gegangen.”
3	Dialekte werden möglichst wortgenau ins Hochdeutsche übersetzt. Wenn keine eindeutige Übersetzung möglich ist, wird der Dialekt beibehalten, z.B.: „Ich gehe heuer auf das Oktoberfest.“	Dialects are translated into standard German as accurately as possible. If no clear translation is possible, the dialect is retained, e.g.: “Ich gehe heuer auf das Oktoberfest.”
4	Umgangssprachliche Partikeln wie „gell, gelle, ne“ werden transkribiert.	Colloquial particles such as “gell, gelle, ne” are transcribed.
5	Stottern wird geglättet bzw. ausgelassen, abgebrochene Wörter werden ignoriert. Wortdoppelungen werden nur erfasst, wenn sie als Stilmittel zur Betonung genutzt werden: „Das ist mir sehr, sehr wichtig.“	Stuttering is smoothed out or omitted, and discontinued words are ignored. Word repetitions are only recorded if they are used as a stylistic device for emphasis: “This is very, very important to me.”
6	Halbsätze, denen die Vollendung fehlt, werden mit dem Abbruchzeichen „/“ gekennzeichnet.	Half-sentences that are incomplete are marked with the break symbol “/”.
7	Interpunktion wird zugunsten der Lesbarkeit geglättet, das heißt, bei kurzem Senken der Stimme oder nicht eindeutiger Betonung wird eher ein Punkt als ein Komma gesetzt. Sinneinheiten sollten beibehalten werden.	Punctuation is smoothed out for readability, i.e., in cases of a brief lowering of the voice or ambiguous emphasis, a period is used rather than a comma. Units of meaning should be retained.

8	Rezeptionssignale wie „hm, aha, ja, genau“, die den Redefluss der anderen Person nicht unterbrechen, werden nicht transkribiert. Sie werden dann transkribiert, wenn sie als direkte Antwort auf eine Frage genannt werden.	Reception signals such as “hm, aha, yes, exactly,” which do not interrupt the other person's flow of speech, are not transcribed. They are transcribed when they are mentioned as a direct response to a question.
9	Pausen ab ca. 3 Sekunden werden durch (...) markiert.	Pauses of approximately 3 seconds or longer are marked by (...).
10	Besonders betonte Wörter oder Äußerungen werden durch VERSALIEN gekennzeichnet.	Words or expressions that are particularly emphasized are indicated by CAPITAL LETTERS.
11	Jeder Sprecherbeitrag erhält eigene Absätze. Zwischen den Sprechern gibt es eine freie, leere Zeile. Auch kurze Einwürfe werden in einem separaten Absatz transkribiert. Mindestens am Ende eines Absatzes werden Zeitmarken eingefügt.	Each speaker's contribution is given its own paragraph. There is a blank line between speakers. Even short interjections are transcribed in a separate paragraph. Time stamps are inserted at least at the end of each paragraph.
12	Emotionale nonverbale Äußerungen der befragten Person und des Interviewers, welche die Aussage unterstützen oder verdeutlichen (wie lachen oder seufzen), werden beim Einsatz in Klammern notiert.	Emotional nonverbal expressions by the interviewee and interviewer that support or clarify the statement (such as laughter or sighs) are noted in parentheses when used.
13	Unverständliche Wörter werden mit „(unv.)“ gekennzeichnet. Längere unverständliche Passagen werden möglichst mit der Ursache versehen: „(unv., Mikrofon rauscht)“. Vermutet man einen Wortlaut, wird die Passage mit einem Fragezeichen in Klammern gesetzt, z.B. „(Axt?)“. Unverständliche Stellen werden mit einer Zeitmarke versehen, wenn innerhalb von einer Minute keine weitere Zeitmarke gesetzt ist.	Unintelligible words are marked with “(unv.)”. Longer unintelligible passages are marked with the cause wherever possible: “(unv., microphone noise)”. If the wording is presumed, the passage is placed in brackets with a question mark, e.g. “(axe?)”. Unintelligible passages are marked with a time stamp if no further time stamp is set within one minute.
14	Die interviewende Person wird durch ein „I:“, die befragte Person durch ein „TN#:“ gekennzeichnet. Die Raute steht für die jeweilige ID, z.B. TN12.	The interviewer is identified by “I:”, and the interviewee by “TN#:”. The hash symbol stands for the respective ID, e.g., TN12.
15	Das Transkript wird als Rich Text Format (RTF-Datei) gespeichert. Die Benennung der Datei erfolgt entsprechend dem Mediendateinamen	The transcript is saved as a Rich Text Format (RTF file). The file is named according to the media file name

	(ohne Endung wav, mp3), beispielsweise: 01_PG_Transkript_TN12.rtf.	(without the wav or mp3 extension), for example: 01_PG_Transcript_TN12.rtf.
16	Sprecherüberlappungen werden mit „//“ gekennzeichnet. Bei Beginn des Einwurfes folgt ein „//“. Der Text, der gleichzeitig gesprochen wird, liegt dann innerhalb dieser „//“ und der Einwurf der anderen Person steht in einer separaten Zeile und ist ebenfalls mit „//“ gekennzeichnet.	Speaker overlaps are marked with “//”. A “//” follows the beginning of the interjection. The text that is spoken simultaneously is then within these “//” and the other person's interjection is on a separate line and is also marked with “//”.
17	Die Partikeln „hm“ werden unabhängig von der Betonung immer „hm“ geschrieben (nicht: „hhhhm“, „mhm“, „hmh“).	The particles “hm” are always written ‘hm’ regardless of emphasis (not: “hhhhm,” “mhm,” “hmh”).
18	Zögerungslaute werden immer „ähm“ geschrieben (nicht: „äm“, „ehm“, „öhm“).	Hesitation sounds are always written as “ähm” (not: “äm,” “ehm,” öhm”).
19	(Maß-) Einheiten werden zur besseren Lesbarkeit nicht ausgeschrieben, z.B. €, %	Units of measurement are not written out in full for better readability, e.g., €, %.
20	Wird in der Aufnahme wörtliche Rede zitiert, wird das Zitat in Anführungszeichen gesetzt: „Und ich sagte dann ‚Na, dann schauen wir mal‘“.	If direct speech is quoted in the recording, the quotation is placed in quotation marks: “And then I said, ‘Well, let's see.’”
21	Auch Redewendungen/Idiome werden wörtlich wiedergegeben, z.B. „über‘s Ohr hauen“ (statt: über das Ohr hauen).	Phrases/idioms are also quoted literally, e.g., “über's Ohr hauen” (instead of: über das Ohr hauen).

References:

Dresing, T. & Pehl, T.: Praxisbuch Interview, Transkription & Analyse. Anleitungen und Regelsysteme für qualitativ Forschende. 8. Auflage. Marburg, 2018.

Kuckartz, U.: Qualitative Inhaltsanalyse. Methoden, Praxis, Computerunterstützung. 5. Auflage. Weinheim, 2022.

Structured Qualitative Content Analysis

Kuckartz, U., & Radiker, S. (2023). Structuring Qualitative Content Analysis. *Qualitative content analysis: Methods, practice and software*.

Phase 1

- Overview
- Exploration of th data
- got familiar with the material

Phase 2

- the following main categories based on the interview guide, literature, and prior knowledge of the interview content (JB interviewed the participants and transcribed most of the interviews) and the app
  - Training
  - Technical functionality
  - Perceived effects
  - Safety
  - App functionality / Ease of use
  - Adherence and motivation
  - Recommendations
  - Requested features / suggestions for improvement
  - Satisfaction
  - "Other"
- Tested applicability on 10-25% of the data → 3 out of 18 interviews
- Main categories were differentiated and rearranged as follows:
  - Prior experience with exercise apps
  - Satisfaction
    - Recommendations
  - Training sessions and exercises
    - Perceived effects
  - Technical functionality
  - Ease of use
  - Adherence and motivation
  - Perceived Safety
  - Other suggestions for improvement

Phase 3

- First coding process
- Three interviews were coded separately by two researchers using the differentiated and rearranged main categories
- Units of meaning are coded
- Differences in code assignments occurred due to
  - Differences in prior knowledge (e.g. regarding technical issues)
  - Two codes were used for the same statement
  - Instead of extending a coded segment, another code was assigned
- Codes were discussed and consensus was reached
- Based on this process, categories and coding guidelines were further defined (see codebook and coding guidelines, p. 12-27)

Phase 4

- Subcategories were built inductively
  - Prior experience with exercise apps
  - Satisfaction

        - Overall satisfaction
        - Continuation of app usage
        - Recommendations
    - Training sessions and exercises
        - Workouts
        - Perceived effects
        - Equipment
        - Exercises
        - Duration
        - Intensity
        - Exercise videos and desciptions
    - Technical functionality
        - Individualization
        - Technical issues
    - Ease of use
    - Adherence and motivation
        - Adherence
        - Motivation
        - Barriers
    - Perceived Safety
    - Other suggestions for improvement
- Definition of subcategories in the codebook

Phase 5

- Second coding process
- Subcategories were employed on first three transcripts
- another two transcripts were coded separately
- The coded segments showed a considerably higher degree of consistency between coders than after the first coding process
- Codes were discussed until consensus was reached
- Categories definitions were further revised to improve differentiation
- One more transcript was coded separately. The assignment of codes to the categories was consistent between coders.
- Categories were considered to be sufficiently defined and differentiated
- Remaining transcripts were coded by JB

Phase 6

- Analysis based on categories
- Export of qualitative case code matrix from MAXQDA
- Quantitative data were added to build a joint display (mixed-methods integration)

Phase 7

- Publication of methods (analysis process) and results

Codebook

Codesystem

Code	Quantity
**1 Prior experience with exercise apps**	21
**2 Satisfaction**	0
2.1 Overall satisfaction	37
2.2 Continuation of app usage	25
2.3 Recommendation	26
**3 Training sessions and exercises**	0
3.1 Workouts	49
3.2 Perceived Effects	52
3.3 Equipment	32
3.4 Exercises	71
3.5 Duration	18
3.6 Intensity	23
3.7 Exercise videos and descriptions	48
**4 Technical functionality**	0
4.1 Individualization	40
4.2 Technical issues	21
**5 Ease of use**	64
**6 Adherence and motivation**	0
6.1 Adherence	16
6.2 Motivation	23
6.3 Barriers	17
**7 Perceived safety**	38
**8 Other suggestions for improvement**	46

Code definitions

1 Prior experience with exercise apps

Description	Experience with health or exercise apps
Application	This code is assigned when interviewees state their prior experience with health or exercise apps
Examples of application	Nein, ich hatte noch keine Erfahrung. Genau. Also auch mit Apps nicht. Was ich ab und zu gemacht hatte, war einfach so auf YouTube mir Videos angeschaut und danach irgendwas mitgemacht. (04_PG_Transkript_TN23, Pos. 2)
Further application	/
Differentiation from other categories	/

2 Satisfaction

2.1 Overall satisfaction

Description	Overall satisfaction with the app
Application	This code is assigned when interviewees mention aspects of their overall satisfaction or feasibility with the app.
Examples of application	• habe ich gerne mit dem Programm trainiert. (04_PG_Transkript_TN23, Pos. 16)<br>• Gesamttraining ist eigentlich in Ordnung. (01_PG_Transkript_TN12, Pos. 19)
Further application	The category is also assigned if interviewees talk about the continuation of the app development<br><br>• *I: Gibt es noch irgendwas, was du mitgeben möchtest?* P25: Ne, außer dass es mich freuen würde, wenn ihr das weiter machen dürftet. Sehr würde ich mich freuen. (06_PG_Transkript_TN25, Pos. 188-189)
Differentiation from other categories	This category is not coded if satisfaction or dissatisfaction refers to aspects related others categories such as exercises, workouts or equipment. In this case the corresponding category is used, e.g.<br>· "jetzt am Ende haben mir die Übungen sehr gut gefallen (04_PG_Transkript_TN23, Pos. 16)"

2.2 Continuation of app usage

Description	Continuation of app usage
Application	This code is assigned when interviewees indicate they would like to continue or discontinue using the app.
Examples of application	• Und das will ich auch wieder benutzen. (01_PG_Transkript_TN12, Pos. 19)
Further application	/
Differentiation from other categories	/

2.3 Recommendation

Description	Recommendation to peers
Application	This code is assigned when interviewees state if they would recommend the app to others.
Examples of application	• *würdest du das Training mit der App weiterempfehlen?* P23: Ja, das habe ich tatsächlich schon gemacht. (04_PG_Transkript_TN23, Pos. 123-124)
Further application	The category is also assigned if interviewees address the suitability or applicability for a certain group. For example: • P36: Auf jeden Fall finde ich aber die App für solche, die eigentlich keinen Sport Angebot haben, finde ich sehr gut. Also wenig Aufwand. Ich brauche keine großen Materialien, ich glaube so ein Theraband hat jeder. Und vielleicht kriegst du dann auch den Sprung, dass sie dann vielleicht etwas MEHR in den Breitensportbereich reingehen, weil viele hast du, die sitzen zu Hause und sehen zu wie der Bauch wächst, // salopp gesagt //. *I: // Trauen sich vielleicht auch nicht richtig* // P36: Also für das Klientel finde ich die App tiptop. (05_PG_Transkript_TN36, Pos. 170-172)
Differentiation from other categories	/

3 Training sessions and exercises

3.1 Workouts

Description	Structure of the exercise sessions
Application	This code is assigned if interviewees evaluate the structure of the exercise sessions or give feedback related to the exercise sessions in general.
Examples of application	• *Wie fandest du sonst die Gestaltung der Trainingseinheiten?* P23: Gut. *I: Also den Aufbau?* P23: Also eigentlich gut, also auch mit diesem / es war ja immer die erste Einheit das Warmup, fünf Minuten, dann zwei so Trainingseinheiten und dann Cooldown. Hat gut gepasst für mich. (04_PG_Transkript_TN23, Pos. 37-40)
Further application	The category is also assigned if interviewees mention unsufficient time for a change of position.
Differentiation from other categories	/

3.2 Perceived Effects

Description	Perceived Effects
Application	This code is assigned when interviewees mention whether they perceived any training effects or not.
Examples of application	• Ne und tatsächlich habe ich auch einen Trainingsfortschritt bei mir persönlich jetzt so subjektiv festgestellt. (04_PG_Transkript_TN23, Pos. 28)
Further application	This category is also assigned if interviewees suggested a reason why there might or might not have been perceived effects, e.g.<br><br>• *Aber hast du das Gefühl, es hat irgendwelche Effekte? Hat es einen Unterschied für dich gemacht? Hat sich was verändert von DA auf jetzt?* P36: Ne, das kann ich ganz schlecht einschätzen und da sind sechs Wochen auch viel zu kurz oder? Um da was zu sehen? *I: Kommt immer ein bisschen drauf an.* P36: Und da ich ja nebenbei auch noch Sport mache. (05_PG_Transkript_TN36, Pos. 165-168)<br><br>• *I: Das heißt, wie hätte das Krafttraining sein sollen, damit du Effekte merkst?* P12: Das müsste etwas intensiver dann sein. (01_PG_Transkript_TN12, Pos. 226-227)<br><br>This code is also assigned when interviewees mention whether the training had an effect on their engagement in physical or sporting activities.

Differentiation from other categories	/

3.3 Equipment

Description	All aspects related to the equipment
Application	This code is assigned if interviewees mention aspects about the used equipment
Examples of application	• · "Was ich von Anfang an sehr schön fand, waren die vielen Varianten mit dem Theraband (04_PG_Transkript_TN23, Pos. 18)"<br>• "*I: Ja, und du hattest eben noch angeschnitten, ähm, ja dass es manchmal ein bisschen tricky war mit der Befestigung des Therabandes?* P12: Ja, das ist eben die Frage. Man muss gucken, wo es nicht reißt, wo man keine scharfen Kanten hat. Und dass es möglichst nicht nach vorne zieht. Also das ist ja speziell bei mir jetzt, das ist ja nicht ganz normal bei mir. (01_PG_Transkript_TN12, Pos. 92-93)"
Further application	The category is also assigned if there are improvement suggestions about the integration of equipment<br><br>• P12: Für mich wäre dann eine Hantel. Wäre ein einfacherer Ausdruck, aber ist ja sowas ähnliches wie das was an der Kugel dran. Ja gut, das ist die Frage. Mit einer Kugel hab ich noch nie gearbeitet, ich arbeite nur mit Hanteln. Kugeln habe ich keine, müsste ich wieder kaufen extra. *I: Aber für dich wäre es schön gewesen, wenn man auch / wenn Hanteln in das Training integriert werden?* P12: Das könnte man gut machen. Wäre eine Idee. (01_PG_Transkript_TN12, Pos. 240-242)
Differentiation from other categories	This category is not coded if the equipment is mentioned in the context of ease of use. In this case, the Category "Ease of Use" is used.<br><br>• P035: Was versteht ihr dann hier unter Box?<br>I: Unter Box? Verstehen wir alles, wo man z.B. / das kann ein Stuhl, eine Stuhloberfläche sein, das kann ein Hocker sein, das kann eine Bank sein, das kann ein Wohnzimmertisch oder alles so auf DER Höhe sein. (03_PG_Transkript_TN35, Pos. 84-85)

3.4 Exercises

Description	All aspects that refer to the exercises as well as the selection or the variety of exercises
Application	This code is assigned if the following aspects are present: feasibility, satisfaction, selection, variety or evaluation of the exercises
Examples of application	• *Waren die Übungen denn soweit passend für dich oder wie würdest du das bewerten?* P12: Die waren soweit passend, wie gesagt so ein bisschen ändern wie man sich hinsetzt im Rollstuhl, wo man das Band festmacht. Da muss man halt gucken, dass es weniger reißt. Das ist halt so. Ansonsten die Übungen an sich funktionieren. (01_PG_Transkript_TN12, Pos. 46-47)<br>• für rein für die Ausdauer fehlt irgendwas. Das sind ja fast alles Kraftübungen und wirklich für die Ausdauer / Nur das kann man meines Erachtens kaum mit dem Gummiband machen, Ausdauer. Und da ist dann Armtrainer, Handbike ähnliches angesagt. Oder viel, viel Rollstuhlfahren. Also da wüsste ich jetzt keine Lösung mit den Bändern. Also als reine Ausdauersache. (01_PG_Transkript_TN12, Pos. 71)<br>• *Hättest du dir noch mehr Abwechslung gewünscht?* P23: Nein, das fand ich eigentlich auch gut gestaltet von der Abwechslung her. (04_PG_Transkript_TN23, Pos. 47-48)
Further application	/
Differentiation from other categories	This category is not coded if an issue with an exercise is due to an individual problem<br><br>• P36: Also ja, ich bin zum Beispiel Th5 und habe eine Einschränkung jetzt an der linken Hüfte. Wenn du dieses Butterfly nach vorne machst, dann drivtest du eben nach rechts ab durch die Hüfte, und dass ist eine Übung, die eben / die eigentlich für mich weniger gut ist. Kann ich jetzt irgendwo den Kopf auflegen, um jetzt den Oberkörper zu entlasten, dann kannst du wieder die Übung ausführen, ohne Probleme. (05_PG_Transkript_TN36, Pos. 8)

3.5 Duration

Description	Duration of the exercise sessions
Application	This code is assigned when interviewees evaluate or state the time it took to complete an exercise session or rate the duration of an exercise session.

Examples of application	• *Dauer der Einheiten, wie würdest du die bewerten? Also der Gesamteinheit jetzt an einem Tag?* P12: Gesamteinheiten sind Ordnung. (01_PG_Transkript_TN12, Pos. 82-83)<br>• Ich habe meistens so plus/-minus 40 Minuten für alles gebraucht. *I: Wie fandest du die Länge, die Dauer? Wäre interessant* P23: Ja, auch gut. Also ich glaube weniger wäre jetzt zu wenig und so war es eigentlich gut, dass ich das schön auch in meinem Tagesablauf einplanen konnte. (04_PG_Transkript_TN23, Pos. 42-44)
Further application	The category is also assigned if interviewees mention whether they could easily integrate the training into their daily routine because of the duration<br>• Und (...) ja, es ist kurz, also es ist überschaubar, man kann es eigentlich gut einbauen so über den Tag oder über die Woche verteilt, weil es ja nicht, weiß nicht, anderthalb Stunden geht, wo man vielleicht schon von vornherein sagt 'oh ne, das das schaffe ich jetzt nicht oder so'. //<br>*I: Also die Dauer der Einheit?* P06: Die Dauer der Einheiten, ja. Ja, das ist so das / also das Positive. (02_PG_Transkript_TN06, Pos. 8-10)
Differentiation from other categories	This category is not coded if the interviewees mentions the duration of the entire intervention period<br>P36: Ne, das kann ich ganz schlecht einschätzen und da sind sechs Wochen auch viel zu kurz oder? (05_PG_Transkript_TN36, Pos. 166)

3.6 Intensity

Description	Intensity of the exercise sessions
Application	This code is assigned when interviewees evaluate the intensity of the exercises and exercise sessions in general
Examples of application	• *Insgesamt von der Intensität her, wie* bewertest du das? P36: Gut, gut. (05_PG_Transkript_TN36, Pos. 93-94)
Further application	/
Differentiation from other categories	The category is not assigned if the statement regarding the intensity of the exercise correlates to perceived effects, e.g.<br>• *I: Das heißt, wie hätte das Krafttraining sein sollen, damit du Effekte merkst?* P12: Das müsste etwas intensiver dann sein. (01_PG_Transkript_TN12, Pos. 226-227)

	The category is also not assigned if resistance of the resistanc band is mentioned. In this case, the category "Equipment" is assigend.<br><br>• I: Ja, wie bist du mit dem Theraband zurecht gekommen?<br>P035: Ja ganz gut. Ganz gut. Ich hatte ein Grünes und Blaues. Das Blaue war das Stärkere, ne?.<br>I: Ja, ja.<br>P035: Das kam mir manchmal schon zu schwach vor. (03_PG_Transkript_TN35, Pos. 91-94)

3.7 Exercise videos and descriptions

Description	All aspects regarding the exercise videos and descriptions
Application	This code is assigned when interviewees give feedback concerning the exercise videos and descriptions as well as the merger of two exercise videos showing different versions of the same exercise.
Examples of application	• P12: Was man nicht ich immer weiß bei einer Übung, ob das jetzt noch an der Brust gezogen wird oder nach hinten gezogen wird, weil dieses Band war mal hier, dieses hier, dieses Strecken so, wo das hier vorne längs läuft oder hinten längs läuft. Das konnte ich selbst auf dem Video nicht erkennen so richtig. Also jedenfalls ich nicht, sag ich mal so. Aber es spielt eigentlich glaube ich weniger eine Rolle oder?. (01_PG_Transkript_TN12, Pos. 143)<br>• P36: Auch das Video in schwarz weiß find ich top. Ja, es lenkt nicht ab. (05_PG_Transkript_TN36, Pos. 144-146)
Further application	This includes suggestions for improvement in this regard.<br><br>• P36: Ja, aber ich bin immer dafür, man sollte vielleicht auch darauf eingehen, dass man es beidseitig macht die Übungen. *I: In den Videos auch?* P36: In den Videos auch. (05_PG_Transkript_TN36, Pos. 30-32)
Differentiation from other categories	This category is not coded if the statement is about safety instructions within the description. In this case, Category "Perceived Safety" is used.<br>• P035: Was auch in dem Video auch drinnen erklärt war, wurde diese Koordinationsübungen hier mit dem Rollstuhl fahren, anheben usw. stand ja auch drinnen, wenn man sich unsicher fühlt, dass man jemanden dazuholen sollte. I: Genau, ja.<br>P035: Also mehr wie darauf hinweisen 'Pass auf, hier

	kann DAS und DAS passieren' könnt ihr ja nicht machen. (03_PG_Transkript_TN35, Pos. 164-166)

4 Technical functionality

4.1 Individualization

Description	Aspects regarding the individualization of the program or the functioning of the algorithm
Application	This code is assigned when interviewees mention aspects in regard to the individualization of the program, the filter options, the algorithm or functions that are regulated by the algorithm such as repetition count.
Examples of application	• P12: Ne, ansonsten läuft das alles, vielleicht die 100 Mal ist ein bisschen lang, muss man bis 100 zählen. *I: Hattest du schon eine mit 100?* P12: Eine hatte ich schon mit 100. (01_PG_Transkript_TN12, Pos. 57-59)
Further application	/
Differentiation from other categories	/

4.2 Technical issues

Description	Report of technical issues
Application	This code is assigned if interviewees mention whether or not there were technical issues
Examples of application	• P36: Du konntest sie öffnen, aber du konntest das Programm nicht aufrufen. *I: Okay. Da stand dann diese Meldung 'Workout konnte nicht geladen werden' oder irgendwie sowas?* P36: Genau, dann bin ich halt zum 1. März und hab am 28. Februar den 1. März gemacht. *I: Ja okay, ja, das war ein Bug, den wir drin hatten, der / den wir behoben haben. Gab es sonst noch irgendwelche technischen Probleme?* P36: Nee, seitdem es wieder hochgeladen wurde gar nicht. (05_PG_Transkript_TN36, Pos. 60-64)"
Further application	/
Differentiation from other categories	This category is not coded if technical issues within the installation process are mentioned. E.g.

	• P36: Ich dachte erst es liegt an mir und dann hab ich die ganze App runtergeschmissen, deinstalliert usw. Dann wieder versucht zu installieren, dann ging gar nichts, weil du ja diesen Einladunglink dringend brauchst. (05_PG_Transkript_TN36, Pos. 188)<br>This category is also not coded if a barrier to use the app was due to technical issues. E.g.<br>• *Gibt es etwas, was dich gehindert hat Mal oder?*P36: Ja, das es nicht gelaufen ist. Das war das Einzige. *I: Das es nicht gelaufen ist, ja gut. Aber ansonsten nicht?* P36: Ne. (05_PG_Transkript_TN36, Pos. 203-206)

5 Ease of use

Description	Aspects regarding ease of use
Application	This code is assigned when interviewees rate ease of use or user friendliness
Examples of application	• *Wie würdest du die Benutzungsfreundlichkeit der App bewerten?* P23: Gut, also kein Problem. *I: Hast du alles da gefunden, wo du es vermutet hast?* P23: Ja genau. (04_PG_Transkript_TN23, Pos. 61-64)<br>• Die App war ansonsten top von der Bedienbarkeit her. (05_PG_Transkript_TN36, Pos. 26)<br>• P12: Und dann geht's los und beim zweiten Mal los weiß man aber nicht mehr, was dann kommt. Da muss man also warten bis das neue Bild kommt. Ach, das muss ich jetzt machen und dann muss ich ja ändern. Und dann muss ich sehen, dass ich mit dem Band irgendwo anders hinkomme. (01_PG_Transkript_TN12, Pos. 25)
Further application	The category is also assigned if the interviewee offers a possible solution to a problem compromising ease of use, e.g.<br>• P12: Ja und dann muss man aber wissen, welche [Übungen] da kommen, ne *I: Okay, also dir würde / oder was würde dir helfen?* P12: Das noch mal anzuzeigen, welche Übung als nächstes wirklich kommt. (01_PG_Transkript_TN12, Pos. 33-35)<br>The category is also assigned if the definition of equipment was unclear:<br>• P035: Was versteht ihr dann hier unter Box?<br>I: Unter Box? Verstehen wir alles, wo man z.B. / das kann ein Stuhl, eine Stuhloberfläche sein, das kann ein Hocker sein, das kann eine Bank sein, das kann ein Wohnzimmertisch oder alles so auf DER Höhe sein. (03_PG_Transkript_TN35, Pos. 84-85)

Differentiation from other categories	This category is not coded if ease of use relates to the installation process<br><br>• P06: Also die Installation war relativ easy. Also es hat im ersten Anlauf ein bisschen gehakt, aber wenn man jetzt eine gewisse Affinität hat mit dem Smartphone und so kriegt man es eigentlich gut hin, würde ich sagen. (02_PG_Transkript_TN06, Pos. 14)

6 Adherence and motivation

6.1 Adherence

Description	Aspects regarding the interviewees' adherence and compliance to the intervention
Application	This code is assigned when interviewees make a statement about their adherence
Examples of application	• *Wie regelmäßig hast du mit der App trainiert?* P36: Ich habe versucht dreimal die Woche. (05_PG_Transkript_TN36, Pos. 197-198)
Further application	The category is also assigned if participants do not use the app accordingly<br>• So, aber dann habe ich mir eben Übungen selber rausgesucht, also was ich tatsächlich gemacht habe, ich habe mir die Übungen alle durchgeguckt und habe dann auch ein paarmal einfach das gemacht, wozu ich Lust hatte. (06_PG_Transkript_TN25, Pos. 39)
Differentiation from other categories	This category is not coded if interviewees state the reason why they did not adhere to the program or mention barriers that impede their adherence. In this case the code "Barriers" is used<br>• P23: Ich habe eigentlich versucht, 2 bis 3 Mal pro Woche zu trainieren. Ich hatte diese eine Phase, wo ich krank war, wo ich fast zwei Wochen nicht trainiert habe. Und dann habe ich noch eine Phase gehabt, da habe ich AUCH nicht trainiert oder weniger trainiert habe. *I: Wann war das denn?* P23: (...) Hier in diesem Zeitraum Ende Februar, Anfang März. Da war ein Todesfall bei uns in der Familie. Ein Onkel von mir ist gestorben. Ja, dann hatte ich noch ein Zahnproblem und es war einfach viel. Also es kamen so ein paar Sachen zusammen, da habe ich eine Zeit lang nicht trainiert. (04_PG_Transkript_TN23, Pos. 100)<br>The category is also not assigned if interviewees state their motivation to participate or adhere to the program. In this case the code "Motivation" is assigned.

	• P23: Nein. Ich habe auch fast, also eigentlich gar nichts anderes gemacht tatsächlich außer der App. Es lag jetzt an der Jahreszeit. Das war jetzt Januar bis März und JETZT kommt der Frühling. Jetzt hole ich mein Rolli Bike raus und werde sicherlich auch draußen viel mehr machen. Aber es war eigentlich eine Zeit, wo es auch für mich schön war, eben diese App nutzen zu können. Also wo ich auch früher schon immer gedacht habe 'oh, jetzt müsste ich was machen'. So gerade Januar, Februar, März und habe mir dann auch teilweise Sachen einfach selbst zusammengestellt habe. Ja, also das hat für mich sehr gut gepasst, dass ich die App nutzen konnte. (04_PG_Transkript_TN23, Pos. 96)

6.2 Motivation

Description	Motivation to participate in the trial or to use the app
Application	This code is assigned when interviewees state their motivation to participate in the trial or to use the app
Examples of application	• P12: Ja, was hat mich dazu motiviert. Ich habe die App da gehabt und dann mach das mal. Ja, es hat mich einfach interessiert, das Ding mal zu probieren. (01_PG_Transkript_TN12, Pos. 258)
Further application	the category is also assigned if participants state, what feature would have motivated them to train:<br>• I: Also hätte man an dem Training selbst auch nichts ändern können, // damit es dir mehr Spaß macht, sondern es lag einfach daran, du machst lieber mit anderen Menschen oder Trainern zusammen? // P035: // Nein, nein. Genau. // Weiß ich nicht. Wenn du positiv die (unv., undeutliche Aussprache), dann kommt ein Tusch oder was weiß ich, ein Konfettiregen auf dem Handy oder / (03_PG_Transkript_TN35, Pos. 135-136)
Differentiation from other categories	/

6.3 Barriers

Description	All aspects regarding barriers to adhering to the exercise program
Application	This code is assigned if the interviewee mentions barriers to adhering to the program

Examples of application	• P23: Ich habe eigentlich versucht, 2 bis 3 Mal pro Woche zu trainieren. Ich hatte diese eine Phase, wo ich krank war, wo ich fast zwei Wochen nicht trainiert habe. Und dann habe ich noch eine Phase gehabt, da habe ich AUCH nicht trainiert oder weniger trainiert habe. Wann war das denn? (...) Hier in diesem Zeitraum Ende Februar, Anfang März. Da war ein Todesfall bei uns in der Familie. Ein Onkel von mir ist gestorben. Ja, dann hatte ich noch ein Zahnproblem und es war einfach viel. Also es kamen so ein paar Sachen zusammen, da habe ich eine Zeit lang nicht trainiert. (04_PG_Transkript_TN23, Pos. 100)
Further application	/
Differentiation from other categories	/

7 Perceived safety

Description	Aspects of perceived safety or need of help
Application	This code is assigned when interviewees state whether they felt safe using the app and performing the suggested exercises or whether they asked for help
Examples of application	• *Hast du dich beim Training sicher gefühlt?* P23: Ja, also ohne Ausnahme. (04_PG_Transkript_TN23, Pos. 93-94)<br>• *Hast du dich dabei sicher gefühlt? Bei den Mobilitätsübungen?* P36: Eigentlich ja. *I: Eigentlich?* P36: Das Einzige ist, wenn man Übungen macht, die über Kopf sind, weil man im Rollstuhl relativ schnell kippt, durch die Wendigkeit. Aber das mache ich ja dann / wenn ich jetzt z.B. mit dem Theraband über Kopf arbeiten muss, dann stelle ich mich direkt an der Wand dran. (05_PG_Transkript_TN36, Pos. 119-122)
Further application	/
Differentiation from other categories	/

8 Other suggestions for improvement

Description	Ideas regarding additional features

Application	This code is assigned when interviewees mention ideas which features they would like to be integrated into the app or which features they think are unnecessary
Examples of application	• P23: Wenn vier Tage nichts gemacht hat, dann bitte Erinnerung oder so. (04_PG_Transkript_TN23, Pos. 122)<br>• P36: Meinst du jetzt gekoppelt mit Ernährungstipps und sowas?<br>*I: Zum Beispiel.*<br>P36: Ah ne, ich denke das ist zu komplex. (05_PG_Transkript_TN36, Pos. 214-216)<br>---<br><br>• P14: Ich finde, es ist diese Schlichtheit. Finde ich auch toll. Es geht wirklich nur um die Übung. Kein Schnick-Schnack, kein 'hast du schon drei Liter Wasser getrunken?' Nebenbei, weißt du ja, die braucht man nicht. Man hat sowieso schon zu viel Input von allem. Und einfach diese Ruhe in dieser App.
Further application	/
Differentiation from other categories	This category is not coded if the additional feature is a possible solution to a before mentioned problem, which was assigned to another code. For example, if the text is assigned to the code "Ease of use", the solution is as well:<br><br>• P12: Ja und dann muss man aber wissen, welche da kommen, ne *I: Okay, also dir würde / oder was würde dir helfen?* P12: Das noch mal anzuzeigen, welche Übung als nächstes wirklich kommt. (01_PG_Transkript_TN12, Pos. 33-35)<br>The category is also not coded if the suggested feature does not target the app but functions of the smartphone. In this case, the segment is not coded, e.g.:<br><br>• P36: Was vielleicht ist, das man vielleicht das Programm wenn das läuft, das man das eventuell auf dem TV spiegeln könnte. (05_PG_Transkript_TN36, Pos. 78)

Coding Guideline

Instructions:

- One coding segment = a statement that is clear outside of its context
- The text passages must be coded into units of meaning, i.e., if a statement spanning several paragraphs is being discussed, for example to clarify or specify questions of understanding, the entire section falls into one coding unit.
- Several aspects of the same statement form a coding unit (e.g., “I was motivated by the fact that ab and cd”).
- Different statements on a topic or code are coded separately

- Coded segments are paraphrased in the code comments (double click on code to add comment)

Example:

Exercise videos and descriptions

Double click on code to add comment

143 P12: Was man nicht ich immer weiß bei einer Übung, ob das jetzt noch an der Brust gezogen wird oder nach hinten gezogen wird, weil dieses Band war mal hier, dieses hier, dieses Strecken so, wo das hier vorne längs läuft oder hinten längs läuft. Das konnte ich selbst auf dem Video nicht erkennen so richtig. Also jedenfalls ich nicht, sag ich mal so. Aber es spielt eigentlich glaube ich weniger eine Rolle oder?. #00:13:30-3#

144 I: Wie / Also es kommt immer auf die Übung an. Ähm, eine Idee, wie man das besser gestalten könnte? #00:13:35-4#

145 P12: Ja spielt das denn so eine große Rolle ob ich jetzt das Band hier hinten längs ziehen oder hier vorne links ziehe? #00:13:42-1#

146 I: Wie gesagt, es kommt auf die Übungen an, ne, aber, hast du eine Idee, wie man das besser gestalten könnte, dass es klarer wird, wie es von uns gemeint ist? #00:13:50-3#

147 P12: Dann müsste das Video deutlicher sein, das ist alles. Ein anderes Band nehmen, was heller ist oder eine andere Farbe hat. #00:13:57-8#

148 I: Also mehr Kontraste?. #00:13:58-2#

149 P12: Weil man kann wirklich nicht erkennen, ob man hier vorne zieht oder über den Rücken zieht. Das kann man nicht erkennen, so unbedingt. #00:14:05-6#

Comment: course of the band is sometimes unclear, a higher contrast would help

- A category is only assigned if a statement is made about the subject of the category. It is not assigned if a signal word is mentioned in a different context (Example 1) or if a question on a topic is not answered (Example 2).

Example 1:

164 I: Ja, Aber waren sonst die Einstellungen zum Beispiel da, wo du sie erwartet hast, also von der Bedienbarkeit? #00:16:22-4#

165 P12: Das ist in Ordnung. #00:16:24-1#

166 I: Hast du alles gefunden oder so 'ach Moment, das finde ich jetzt nicht' oder musstest du irgendwann mal suchen? #00:16:28-2#

167 P12: Das waren nur die ersten zwei Übungen, da wo ich dann gefragt habe 'wie geht das', was ich nicht verstanden hatte zuerst. Weil ... ja Workout, und dann ist, ja, und dann geht es aber doch noch weiter, weil das Teil eins ist. Aber ansonsten / #00:16:45-0#

168 I: Aber jetzt auch so zu den / du kannst ja einstellen, welches Equipment habe ich, da ist jetzt noch nicht so viel hinterlegt, weil wir ja noch in der Pilotphase sind. Aber welches Equipment habe ich, welche Bewegungen kann ich machen und welche nicht? Diese Einstellung, hast du das genutzt? #00:16:58-0#

169 P12: Die habe ich benutzt. Ich habe vieles rausgeschmissen. #00:17:00-0#

Equipment is mentioned, but it is not about the equipment itself, but rather about the filter settings and whether they were easy to find.

Example 2:

161 I: Okay, super. Dann haben wir da ja gar nicht mehr so viel dran zu drehen. Hast du das Gefühl durch das Training mit der App hat sich irgendetwas verändert?

..Motivation

162 P36: Na, du bist schon gezwungen, das du was machen musst. Ich mache sonst zwar auch viel. Also ich gehe immer zur Physio und auch zum Sport, aber ich finde es so als Ergänzung dazu schön. Und wenn du es machst hast du eine Anleitung, wie ich vorhin schon gesagt habe. Das ist nicht, dass du jetzt ran an den Sport gehst und denkst 'was machst du heute?' Sondern du siehst, aha so sieht das aus.

The interviewee is asked about training effects, but does not respond to the question. The code “perceived effects” is not coded.
